# Genome-wide association study identifies genomic regions associated with key reproductive traits in Korean Hanwoo cows

**DOI:** 10.1186/s12864-024-10401-3

**Published:** 2024-05-23

**Authors:** Md Azizul Haque, Yun-Mi Lee, Jae-Jung Ha, Shil Jin, Byoungho Park, Nam-Young Kim, Jeong-Il Won, Jong-Joo Kim

**Affiliations:** 1 Department of Biotechnology, Yeungnam University, Gyeongsan, Gyeongbuk 38541 Korea; 2 Gyeongbuk Livestock Research Institute, Yeongju, 36052 Korea; 3 Hanwoo Research Institute, National Institute of Animal Science, Pyeongchang, 25340 Korea

**Keywords:** Candidate gene, Genome-wide association study, Hanwoo, Reproductive traits

## Abstract

**Background:**

Conducting genome-wide association studies (GWAS) for reproductive traits in Hanwoo cattle, including age at first calving (AFC), calving interval (CI), gestation length (GL), and number of artificial inseminations per conception (NAIPC), is of paramount significance. These analyses provided a thorough exploration of the genetic basis of these traits, facilitating the identification of key markers for targeted trait improvement. Breeders can optimize their selection strategies, leading to more efficient and sustainable breeding programs, by incorporating genetic insights. This impact extends beyond individual traits and contributes to the overall productivity and profitability of the Hanwoo beef cattle industry. Ultimately, GWAS is essential in ensuring the long-term genetic resilience and adaptability of Hanwoo cattle populations. The primary goal of this study was to identify significant single nucleotide polymorphisms (SNPs) or quantitative trait loci (QTLs) associated with the studied reproductive traits and subsequently map the underlying genes that hold promise for trait improvement.

**Results:**

A genome-wide association study of reproductive traits identified 68 significant single nucleotide polymorphisms (SNPs) distributed across 29 *Bos taurus* autosomes (BTA). Among them, BTA14 exhibited the highest number of identified SNPs (25), whereas BTA6, BTA7, BTA8, BTA10, BTA13, BTA17, and BTA20 exhibited 8, 5, 5, 3, 8, 2, and 12 significant SNPs, respectively. Annotation of candidate genes within a 500 kb region surrounding the significant SNPs led to the identification of ten candidate genes relevant to age at first calving. These genes were: *FANCG*, *UNC13B*, *TESK1*, *TLN1*, and *CREB3* on BTA8; *FAM110B*, *UBXN2B*, *SDCBP*, and *TOX* on BTA14; and *MAP3K1* on BTA20. Additionally, *APBA3*, *TCF12*, and *ZFR2*, located on BTA7 and BTA10, were associated with the calving interval; *PAX1*, *SGCD*, and *HAND1*, located on BTA7 and BTA13, were linked to gestation length; and *RBM47*, *UBE2K*, and *GPX8*, located on BTA6 and BTA20, were linked to the number of artificial inseminations per conception in Hanwoo cows.

**Conclusions:**

The findings of this study enhance our knowledge of the genetic factors that influence reproductive traits in Hanwoo cattle populations and provide a foundation for future breeding strategies focused on improving desirable traits in beef cattle. This research offers new evidence and insights into the genetic variants and genome regions associated with reproductive traits and contributes valuable information to guide future efforts in cattle breeding.

**Supplementary Information:**

The online version contains supplementary material available at 10.1186/s12864-024-10401-3.

## Background

Hanwoo cattle, an indigenous breed of Korea, is a testament to the nation’s rich agricultural heritage. Revered for their exceptional qualities and cultural significance, they transcend being mere livestock; they embody a living tradition deeply connected to the country’s history and traditions. This connection reflects the harmonious balance between traditional farming practices and contemporary agricultural methods. Emerging from the lush landscapes of the Korean Peninsula, these cattle symbolize national pride, celebrated for their gentle disposition and adaptability to local climates and, notably, for the superior quality of their beef [1].

Genetic improvement efforts within Hanwoo cattle breeding programs have historically prioritized carcass quality and growth traits, driven by the accessibility of trait information and the simplicity of analytical techniques [2]. This focus is justified by the significant economic importance of these traits [3]. However, a notable shift has occurred in recent years, with increased attention being directed toward the genetic analyses of reproductive traits in Hanwoo cattle. This shift was driven by the recognition that reproductive traits are important in calf productivity. These traits carry substantial economic significance for sustainable food production, particularly in the context of monotonic livestock, such as cattle and buffaloes. Prioritizing fertility enhancement has emerged as the optimal strategy for mitigating culling costs, preserving valuable genetic resources, and augmenting overall farm profitability [4]. Historically, challenges in improving reproductive efficiency have been attributed to factors such as low heritability, the binomial nature of a short-controlled breeding season, and delayed expression of traits throughout an animal’s life [5]. This evolution from carcass to reproductive traits underscores the need for comprehensive and sustainable Hanwoo cattle management practices.

The key reproductive traits under the current study included age at first calving (AFC), calving interval (CI), gestation length (GL), and number of artificial inseminations per conception (NAIPC). These traits play pivotal roles in determining the efficiency and effectiveness of cattle breeding programs. AFC is an important trait representing the age at which a female bovine, typically a heifer, gives birth to its first calf [6]. The optimal age for first calving varies among cattle breeds and is influenced by factors such as genetics, nutrition, and management practices [7]. Generally, AFC strikes a balance between allowing heifers to reach sufficient maturity for a healthy pregnancy and ensuring timely reproductive efficiency. For many cattle breeds, the recommended age for first calving is between 20 and 27 months [8]. Breeders aim to achieve optimal AFC to ensure that heifers are adequately developed and capable of handling the physical demands of pregnancy and lactation. Achieving this requires proper nutrition, well-managed breeding programs, and attention to health and growth factors. Heifers that calve at an appropriate age contribute to the overall efficiency of breeding programs, leading to healthier and more resilient cattle populations [9]. Breeders use careful monitoring and management practices to achieve optimal AFC, thereby promoting long-term success in their cattle breeding endeavors.

Calving interval, a vital reproductive trait, represents the duration between consecutive calvings in a female bovine reproductive cycle. This key factor directly influences the frequency and regularity of calving within a herd, affecting overall reproductive efficiency and productivity. The optimal calving interval varies according to specific breeding goals, management practices, and cattle breed characteristics. Generally, a shorter calving interval, typically ranging from 12 to 13 months, is preferred [10]. Achieving an efficient calving interval is crucial for maintaining a consistent calving pattern, optimizing the herd’s reproductive performance, and ensuring a steady production cycle. Multiple factors influence the calving interval, including the postpartum recovery period, genetics, nutrition, and overall herd management practices [11]. The ability of cows to swiftly recover after calving, combined with proper nutrition and breeding strategies, contributes to shorter calving intervals. Efficient management of the calving interval is essential to sustain productive and economically viable cattle breeding programs. Breeders focus on implementing sound reproductive management practices, such as effective breeding protocols and proper healthcare, to minimize the calving interval and enhance the overall reproductive efficiency of the herd [12]. A well-maintained calving interval ensures a steady supply of calves and contributes to the success and sustainability of cattle breeding.

Gestational length, another vital reproductive parameter in cattle breeding, refers to the duration of pregnancy from conception to the calf’s birth. The optimal gestation duration varies among cattle breeds and generally ranges from 279 to 287 days [13]. Effective management of gestation length is key to ensuring a successful calving process, minimizing the risk of complications, and promoting the overall well-being of both the dam (the pregnant cow) and the newborn. Cattle breeds can exhibit variations in inherent gestation length and selective breeding can be used to achieve the desired traits [14]. Understanding and monitoring gestation length are essential components of successful reproductive management programs for breeders. Proper nutrition during pregnancy, routine veterinary care, and attention to environmental factors contribute to maintaining an optimal gestation length [15]. Effective management of gestation length enhances the chances of a healthy and trouble-free calving process, ensuring the health of the cow and the newborn calf.

The number of artificial inseminations per conception is a key measure in cattle breeding that reflects the efficiency of the artificial insemination (AI) process. This signifies the number of attempts, typically through artificial insemination, required for female bovines to conceive successfully. Achieving optimal NAIPC is essential for maximizing fertility and conception efficiency in cattle breeding programs. A lower NAIPC indicates a higher conception rate, suggesting that fewer insemination attempts are needed to achieve a successful pregnancy [16]. Conversely, a higher NAIPC may suggest challenges in fertility or other factors affecting conception [17]. Several factors influence NAIPC, including the reproductive health of the female, the timing and technique of insemination, the quality of semen used, and overall herd management practices [18]. Breeders aim to minimize NAIPC through careful monitoring of the estrous cycle, proper timing of insemination, and the use of high-quality semen from proven sires [18–20]. By focusing on this parameter, breeders can implement targeted strategies to optimize fertility, reduce the number of insemination attempts, and improve the overall effectiveness of cattle breeding programs [21]. The efficient management of NAIPC aligns with the broader goals of sustainable breeding practices and contributes to the long-term success of the cattle industry.

The sequencing of the bovine genome in 2009, along with advancements in information technology, facilitated the development of modern, scientifically designed breeding schemes for enhancing economically important traits. These efforts resulted in a notable increase in both the quality and quantity of milk and meat production per animal [22]. The GWAS on Hanwoo female reproductive traits holds significant importance for addressing future challenges and advancing breeding strategies. Understanding the genetic basis of reproductive performance can help identify key genomic regions associated with important traits, providing valuable information on the underlying genetic factors influencing female reproductive characteristics. The identification of essential genes, haplotypes, and their regulatory mechanisms as markers for quantitative traits has the potential to enhance strategies for selecting beef cattle both currently and in the future [22]. There has been a significant focus on conducting genome-wide association studies (GWAS) to investigate economic traits in cattle, including fertility traits [23–28], production traits [29–31], somatic cell score [32–35], and disease resistance [36–39] in recent years. Numerous significant single nucleotide polymorphisms (SNPs) and biologically relevant genes were identified. However, a limited number of studies with sample sizes exceeding ~ 3000 individuals focus on beef cattle reproductive traits [24, 27, 40, 41]. Therefore, there is a need to revisit GWAS for reproductive traits using a larger population sample to obtain more reliable and comprehensive results. Despite research on genetic parameter estimations [2] and the accuracy of genomic predictions for reproductive traits [42] in recent years, GWAS in the context of Hanwoo cattle remains an underexplored area, with no notable studies addressing these reproductive traits. The scarcity of literature addressing the GWAS for Hanwoo reproductive traits prompted the primary objective of our current investigation. In our study, we analyzed a population of over 10,000 individuals, representing a significant improvement in terms of sample size compared with that of previous studies. Exploring the genetic architecture of these reproductive traits within this breed would provide baseline information regarding the quantitative trait loci (QTLs) or genes underlying the traits under investigation. Therefore, our objectives were to identify significant SNPs associated with these traits, explore the genetic architecture and biological relevance of these markers at the whole-genome level, and identify potential candidate genes in Hanwoo cattle.

## Results

### Phenotypes

**Fig. 1:**
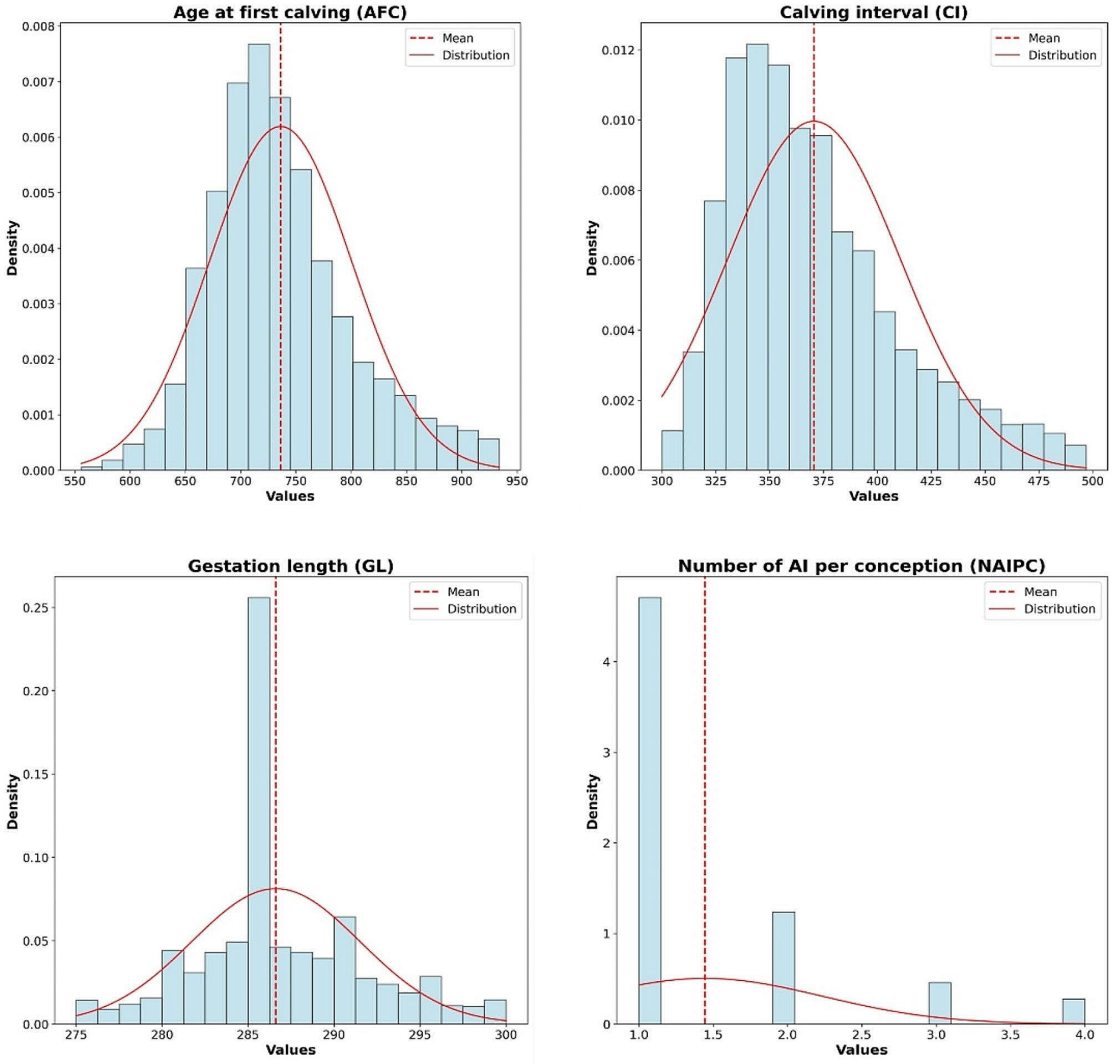
Distribution of AFC, CI, GL, and NAIPC phenotype in Hanwoo cows

Prior to conducting any statistical analysis, the phenotypic data of AFC, CI, and GL were checked for normality through visualization of the data distribution and application of the Kolmogorov-Smirnov Normality Test (Fig. 1). The results revealed that the data of AFC, CI, and GL followed a normal distribution. Additionally, records of animals with an NAIPC above four were excluded from the dataset, as visualized in Fig. 1. Table 1 summarizes the key reproductive traits in the Hanwoo cattle population considered in our GWAS, focusing on animals with both genotypic and phenotypic records after quality control. The mean AFC duration was 736.18 days, and the standard deviation was 64.43 days, indicating a moderate level of variability. The CI displayed a mean of 370.56 days and a standard deviation of 40.04 days, suggesting a relatively consistent pattern. The mean duration was 286.62 days, with a narrow standard deviation of 4.91 days, indicating a more homogeneous distribution. The NAIPC, measured on a scale of 1 to 4, had a mean value of 1.44 and a standard deviation of 0.79, demonstrating a moderate level of variability in this reproductive parameter.

**Table 1:** Summary of the statistics of the Hanwoo reproductive traits used in the GWAS

Traits	Number of animals	Mean	SD	Minimum	Maximum
AFC (days)	10,148	736.18	64.43	556	934
CI (days)	7,994	370.56	40.04	300	497
GL (days)	10,426	286.62	4.91	275	303
NAIPC (1–4)	11,204	1.44	0.79	1	4

SD, standard deviation; AFC, age at first calving; CI, calving interval; GL, gestation length; NAIPC, number of artificial inseminations per conception.

### SNP genotyping

**Fig. 2:**
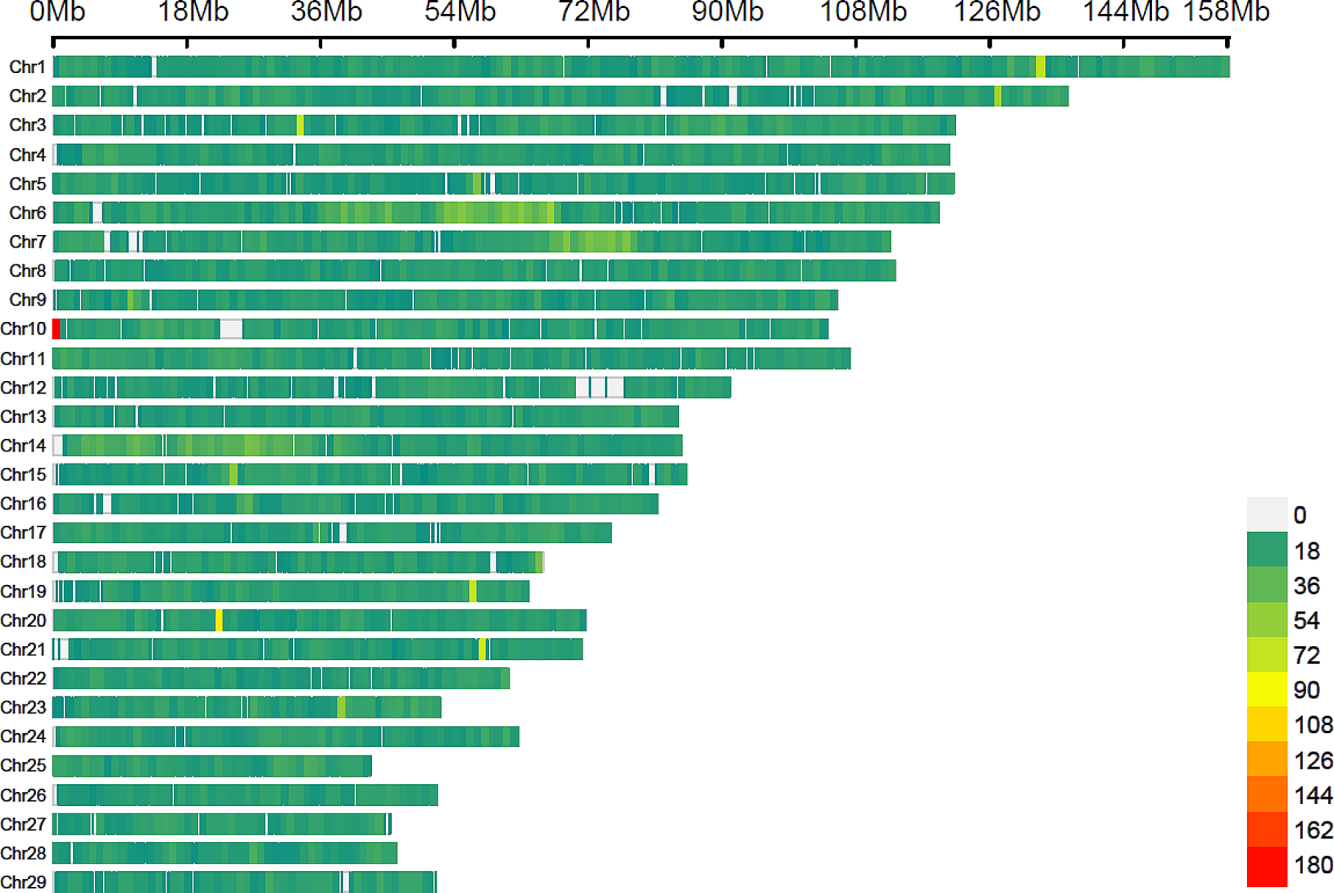
Distribution of SNPs across chromosomes after quality control

After performing quality control procedures involving 78.30% of the initial SNPs across all 29 *Bos taurus* autosomes (BTA), a set of 40,807 common SNPs was selected. The distribution of these markers was uneven, with a significant over-representation of specific chromosomes, as shown in Fig. 2. Among these, BTA1 had the highest number of SNPs (2570), covering approximately 158 megabases (Mb), whereas BTA28 had the fewest SNPs (705), spanning approximately 46.1 Mb. Additionally, BTA1, BTA2, BTA3, and BTA6 harbored more than 2000 SNPs each.

### Population structure and linkage disequilibrium (LD) decay analysis

**Fig. 3:**
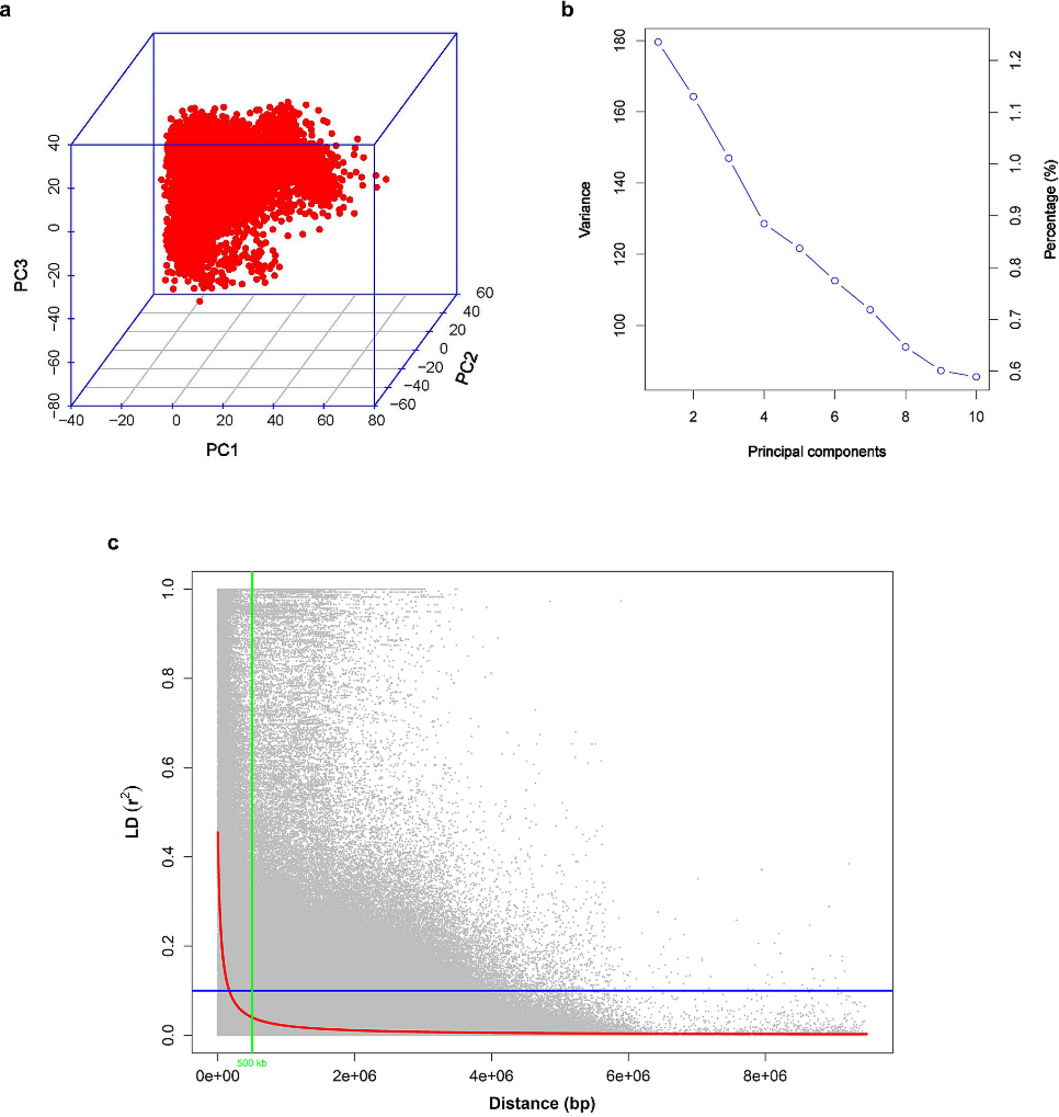
Analysis of population structure in Hanwoo cows using quality-controlled SNP data. (**a**) Principal component analysis (PCA) plot. (**b**) Scree plot illustrating variance accumulation of the top ten principal components (PCs). The x-axis denotes the top ten PCs, while the y-axis represents eigenvalues indicating the amount of variation. Accumulated variance for each PC is marked by an empty circle, and the “elbow” point indicates the number of factors to generate. (**c**) Genome-wide linkage disequilibrium (LD) decay plot. LD, measured as the squared correlation coefficient (r^2^) between pairs of polymorphic markers, is plotted against their genetic distance (bp) across the chromosomes. The red line represents the moving average of the ten adjacent markers, with each gray dot signifying the distances between two markers and their corresponding squared correlation coefficient. The blue line marks the LD cutoff of 0.1, while the green line signifies the critical LD at a distance of less than 500 kb

This study used principal component analysis (PCA) to explore the genetic structure of Hanwoo cattle using quality-controlled SNP data. This analysis revealed a clear genotype cluster in the dataset, as illustrated in Fig. 3a. The scree plot of the eigenvalues demonstrated that the first two principal components explained 2.3% of the total variability (Fig. 3b). The square of the correlation coefficient between markers at two loci (r^2^) was used to estimate LD. It is generally expected that LD will decay by half when the r^2^ value falls below 0.2 [43]. The LD decayed to an r^2^ value of 0.08 at approximately 500 kb in the Hanwoo cattle population under investigation (Fig. 3c).

### Association analysis

GWAS of reproductive traits revealed 68 significant SNPs distributed across 29 BTA. However, the distribution was uneven, with specific chromosomes exhibiting more SNPs associated with each trait. Notably, BTA14 had the highest number of identified SNPs, totaling 25. Additionally, BTA6, BTA7, BTA8, BTA10, BTA13, BTA17, and BTA20 displayed 8, 5, 5, 3, 8, 2, and 12 significant SNPs, respectively. A comprehensive summary of the GWAS results is provided, including significant SNP IDs for the studied traits, SNP positions on the respective BTAs, allele types (minor/major), minor allele frequency (MAF), p values, and nearby candidate genes.

### Marker loci associated with AFC

**Fig. 4:**
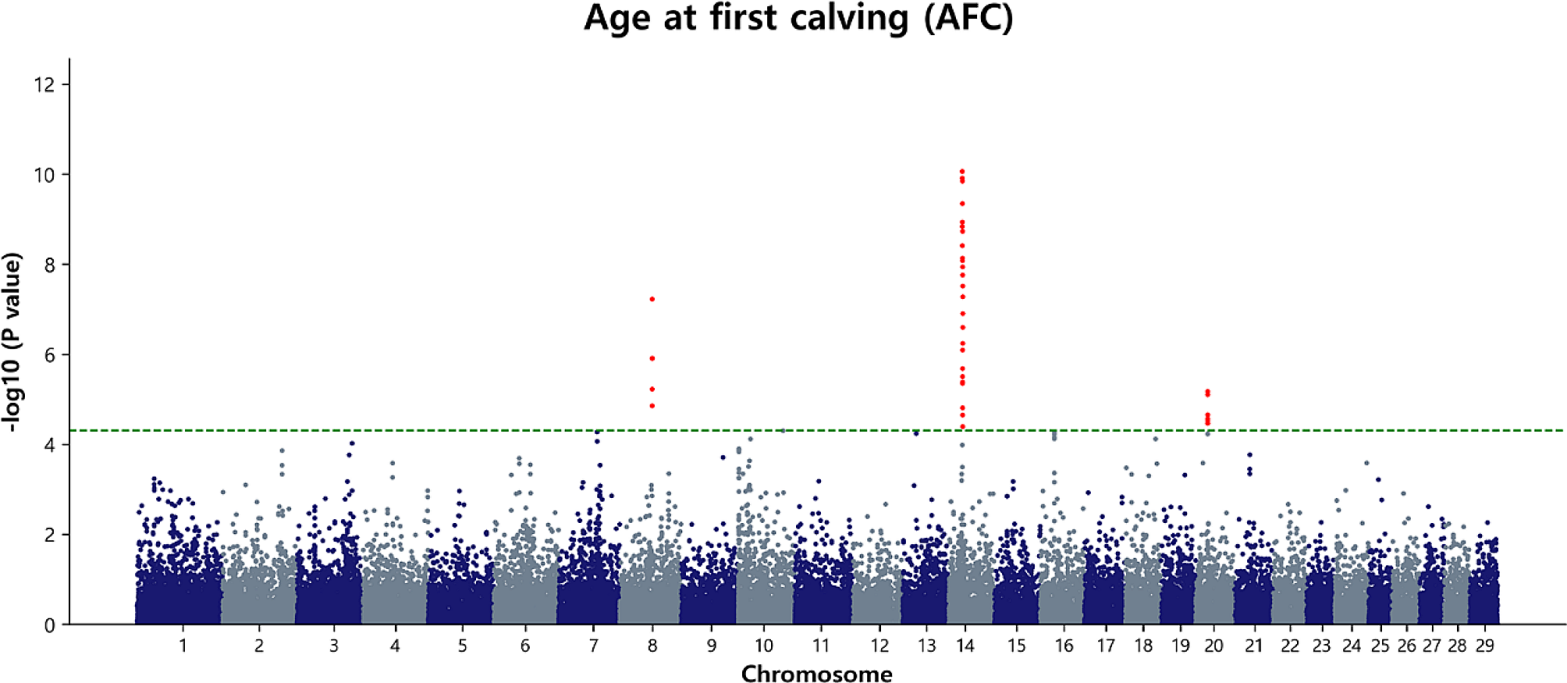
Manhattan plot of GWAS for AFC in Hanwoo cows. The y-axis represents -log_10_ (observed) p-values for genome-wide SNPs against their respective positions on each chromosome (x-axis). The horizontal green line indicates the suggestive (5 × 10^− 5^) threshold level

GWAS results for AFC in Hanwoo cattle revealed a diverse set of SNPs associated with this reproductive trait. In total, 38 SNPs were identified across all 29 BTA (Table 2; Fig. 4). Notably, BTA14 exhibited the highest number of SNPs (25 variants), indicating a potential genomic hotspot for genetic factors influencing AFC. Five SNPs were identified on BTA8, and these genetic markers spread across a genomic range with positions ranging from 59.91 Mb to 60.16 Mb. Similarly, BTA14 emerges as a prominent genomic region containing a substantial number of SNPs associated with AFC. These SNPs spanned a range of positions from 26.16 Mb to 27.16 Mb, underlining the extensive genetic landscape on BTA14 that influences the age at which Hanwoo cattle experience their first calving. Additionally, BTA20 featured in the GWAS results, with a distinct set of eight SNPs associated with AFC, positioned between 22.17 Mb and 22.20 Mb.

**Table 2:** Genome-wide significant SNPs underlying AFC in Hanwoo cows

SNP ID	BTA	Position (bp)	Allele	MAF	*p*-value	Gene
ARS-USMARC-Parent-AY850194-no-rs	8	59,996,431	[T/C]	0.442	6.00 × 10^− 8^	*UNC13B*, *LOC516849*, *RUSC2*, *LOC507810*
ARS-BFGL-NGS-84,004	8	60,158,030	[A/C]	0.345	1.23 × 10^− 6^	*UNC13B*, *LOC516849*, *RUSC2*, *LOC507810*, *TESK1*, *CD72, SIT1*, *CCDC107*, *C9orf100*, *CA9*, *TPM2*, *TLN1*, *LOC100297528*, *CREB3*, *GBA2*, *RGP1*, *LOC100295064*
ARS-BFGL-NGS-112,343	8	60,037,216	[A/G]	0.345	1.25 × 10^− 6^	*UNC13B*, *LOC516849*, *RUSC2*, *LOC507810*, *TESK1*, *CD72*
BTB-00350148	8	59,905,644	[G/A]	0.363	5.98 × 10^− 6^	*LOC100137826*, *VCP*, *FANCG*, *PNN*, *PIGO*, *STOML2*, *KIAA1539*, *UNC13B*, *LOC516849*
Hapmap53279-rs29026049	8	59,954,021	[G/A]	0.383	1.40 × 10^− 5^	*FANCG*, *PNN*, *PIGO*, *STOML2*, *KIAA1539*, *UNC13B*, *LOC516849*, *RUSC2*
rs42304778	14	26,162,492	[G/A]	0.221	8.73 × 10^− 11^	*FAM110B*, *LOC100295254*, *UBXN2B*, *LOC510507*
rs42304792	14	26,173,037	[T/C]	0.221	1.23 × 10^− 10^	*FAM110B*, *LOC100295254*, *UBXN2B*, *LOC510507*
rs41725705	14	26,570,145	[A/T]	0.224	1.43 × 10^− 10^	*SDCBP*, *NSMAF*, *TOX*
rs209439851	14	26,443,481	[T/A]	0.225	4.52 × 10^− 10^	*UBXN2B*, *LOC510507*, *SDCBP*, *NSMAF*, *TOX*
rs43083563	14	26,311,106	[A/T]	0.224	1.16 × 10^− 9^	*FAM110B*, *LOC100295254*, *UBXN2B*, *LOC510507*, *SDCBP*, *NSMAF*
rs110634307	14	26,223,753	[T/C]	0.224	1.46 × 10^− 9^	*FAM110B*, *LOC100295254*, *UBXN2B*, *LOC510507*
rs41627959	14	26,804,892	[C/T]	0.223	1.87 × 10^− 9^	*TOX*
BTB-01143580	14	26,264,142	[A/G]	0.224	3.88 × 10^− 9^	*FAM110B*, *LOC100295254*, *UBXN2B*, *LOC510507*, *SDCBP*, *NSMAF*
rs41725159	14	26,619,895	[A/C]	0.224	7.36 × 10^− 9^	*SDCBP*, *NSMAF*, *TOX*
rs41725162	14	26,621,673	[T/C]	0.224	8.36 × 10^− 9^	*SDCBP*, *NSMAF*, *TOX*
rs42406058	14	26,848,418	[G/A]	0.223	1.15 × 10^− 8^	*TOX*
rs42406039	14	26,859,737	[T/A]	0.223	1.75 × 10^− 8^	*TOX*
rs42404009	14	27,030,664	[A/C]	0.223	3.06 × 10^− 8^	*TOX*
rs42403994	14	27,109,032	[T/G]	0.223	5.31 × 10^− 8^	*TOX*
rs42403958	14	27,139,604	[T/C]	0.223	1.25 × 10^− 7^	*TOX*
Hapmap27934-BTC-065223	14	27,155,254	[C/T]	0.223	2.55 × 10^− 7^	*TOX*, *CA8*
rs132748716	14	27,050,379	[A/G]	0.223	5.78 × 10^− 7^	*TOX*, *CA8*
Hapmap30932-BTC-011225	14	26,766,010	[T/C]	0.223	8.09 × 10^− 7^	*TOX*
rs41724028	14	26,776,546	[G/A]	0.223	2.09 × 10^− 6^	*TOX*
rs41724548	14	26,743,126	[C/A]	0.224	3.15 × 10^− 6^	*TOX*
rs41724547	14	26,746,062	[C/T]	0.224	4.15 × 10^− 6^	*TOX*
rs109374728	14	26,656,398	[A/T]	0.225	4.49 × 10^− 6^	*NSMAF*, *TOX*
UA-IFASA-7902	14	26,800,529	[T/C]	0.381	1.56 × 10^− 5^	*TOX*
Hapmap31672-BTC-065429	14	26,836,013	[T/C]	0.380	2.26 × 10^− 5^	*TOX*
rs208481177	14	26,995,868	[A/G]	0.244	4.07 × 10^− 5^	*TOX*
BovineHD2000006657	20	22,201,747	[C/A]	0.075	6.77 × 10^− 6^	*GPBP1*, *LOC524269*, *MIER3*, *LOC540253*, *MAP3K1*
BovineHD2000006652	20	22,195,752	[T/C]	0.075	7.93 × 10^− 6^	*GPBP1*, *LOC524269*, *MIER3*, *LOC540253*, *MAP3K1*
BovineHD2000006664	20	22,209,047	[G/A]	0.071	2.23 × 10^− 5^	*GPBP1*, *LOC524269*, *MIER3*, *LOC540253*, *MAP3K1*
BovineHD2000006653	20	22,197,248	[C/T]	0.071	2.73 × 10^− 5^	*GPBP1*, *LOC524269*, *MIER3*, *LOC540253*, *MAP3K1*
BovineHD2000006655	20	22,200,130	[G/A]	0.071	3.01 × 10^− 5^	*GPBP1*, *LOC524269*, *MIER3*, *LOC540253*, *MAP3K1*
BovineHD2000006648	20	22,173,199	[A/G]	0.071	3.43 × 10^− 5^	*GPBP1*, *LOC524269*, *MIER3*, *LOC540253*, *MAP3K1*
BovineHD2000006659	20	22,203,443	[A/G]	0.071	3.43 × 10^− 5^	*GPBP1*, *LOC524269*, *MIER3*, *LOC540253*, *MAP3K1*
BovineHD2000006665	20	22,210,409	[A/C]	0.071	3.43 × 10^− 5^	*GPBP1*, *LOC524269*, *MIER3*, *LOC540253*, *MAP3K1*

SNP, single nucleotide polymorphism; BTA, *Bos taurus* autosome; bp, base pair; MAF, minor allele frequency. The reference genome assembly utilized for GWAS is Bos_taurus_UMD_3.1.1.

**Fig. 5:**
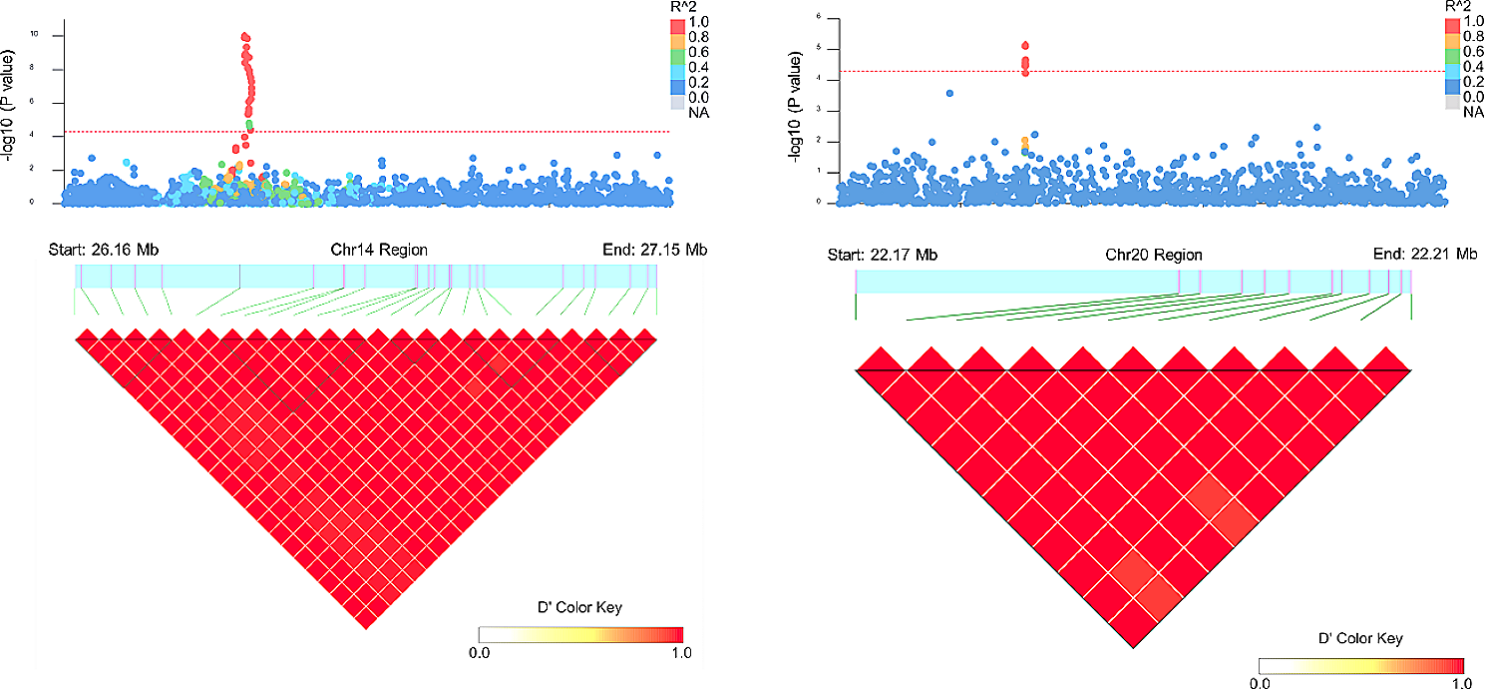
Regional association plot (top) showing the distribution of significant loci associated with AFC at various positions on BTA14 and BTA20 and heatmap of LD (bottom). The red horizontal line indicates -log_10_P = 4.30

The identified SNPs on BTA20 exhibited complete LD and were located within a 38.76 kb haplotype block (20: 22,173,199–22,211,958 bp). This suggested that mutations near the potential QTL may have a significant effect on AFC (Fig. 5). The BTA8 regional association plot and heatmap of LD are presented in (Additional file 1: Fig. S1). Furthermore, among these SNPs, the highest significance was observed at position 26.16 Mb on BTA14 (rs42304778, *p* = 8.73 × 10^− 11^). Additionally, apart from rs42304778, other noteworthy SNPs on BTA14 based on the p-value threshold included rs42304792, *p* = 1.23 × 10^− 10^; rs41725705, *p* = 1.43 × 10^− 10^; rs209439851, *p* = 4.52 × 10^− 10^; rs43083563, *p* = 1.16 × 10^− 9^; and rs110634307, *p* = 1.46 × 10^− 9^, located on BTA14 at the position of 26.17 Mb to 26.57 Mb (Fig. 4). The genomic inflation factor (λ) was calculated to assess population stratification, revealing that AFC exhibited a λ value of 0.996 (Additional file 2: Fig S2a). To visually represent the observed versus expected p-values (-log_10_P) for AFC, we generated quantile-quantile (QQ) plots (Additional file 2: Fig S2a). The QQ plots in this study clearly demonstrated a close alignment between the observed and expected values, indicating that the p-values were normally distributed. This alignment suggested that population stratification was effectively addressed using the appropriate model, thereby enhancing the likelihood of identifying true associations.

### Marker loci associated with CI

**Fig. 6:**
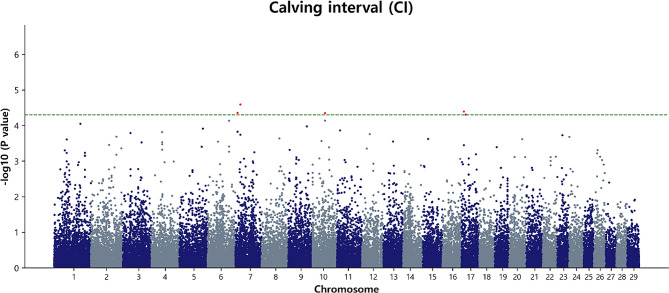
Manhattan plot of GWAS for CI in Hanwoo cows. The y-axis represents -log_10_ (observed) p-values for genome-wide SNPs against their respective positions on each chromosome (x-axis). The horizontal green line indicates the suggestive (5 × 10^− 5^) threshold level

The GWAS revealed five significant chromosomal regions in the calving interval of Hanwoo cattle (Table 3; Fig. 6). Specifically, BTA7 featured two identified SNPs located in genomic regions of 9.56 Mb and 21.57 Mb, respectively. Additionally, within the GWAS results, BTA10 featured a noteworthy SNP associated with AFC at 53.11 Mb. Furthermore, two SNPs on BTA17, positioned at 10.12 Mb and 18.32 Mb, exhibited significance with the calving interval. Among the notable findings, the significant SNPs associated with this trait were BovineHD0700002480 (*p* = 4.42 × 10^− 5^), Hapmap33901-BES9_Contig395_449 (*p* = 2.58 × 10^− 5^), BTB-00425619 (*p* = 4.50 × 10^− 5^), NIAS_SPC_00646 (*p* = 4.07 × 10^− 5^), and ARS-BFGL-NGS-11,930 (*p* = 4.95 × 10^− 5^), respectively. The regional association plot and LD heat map for the BTA7, BTA10, and BTA17 regions are presented in (Additional file 1: Fig S1). In addition, the study used a QQ plot that revealed a close alignment between the observed and expected p-values. The λ was calculated and found to be 1.021, indicating that potential confounding factors, such as population stratification, were adequately addressed (Additional file 2: Fig S2b).

**Table 3:** Genome-wide significant SNPs underlying CI in Hanwoo cows

SNP ID	BTA	Position (bp)	Allele	MAF	*p*-value	Gene
BovineHD0700002480	7	9,558,112	[A/C]	0.239	4.42 × 10^− 5^	*LOC787503*, *LOC787540*, *LOC504888*, *LOC787574*, *LOC787604*, *LOC100337378*, *LOC616989*, *LOC783492*, *LOC100299890*, *LOC616964*, *LOC787665*
Hapmap33901-BES9_Contig395_449	7	21,574,886	[G/A]	0.064	2.58 × 10^− 5^	*ZFR2*, *MATK*, *RAX2*, *MRPL54*, *APBA3*, *TJP3*, *PIP5K1C*, *LOC528952*, *LOC100295917*, *TBXA2R*, *LOC100336987*, *LOC100336995*, *GIPC3*, *HMG20B*, *MGC137027*, *LOC526668*, *LOC100337014*, *FZR1*, *LOC100295989*, *DOHH*, *LOC510079*, *LOC618071*, *NFIC*, *LOC100337236*
BTB-00425619	10	53,110,990	[C/T]	0.074	4.50 × 10^− 5^	*LOC517509*, *TCF12*
NIAS_SPC_00646	17	10,124,524	[T/C]	0.104	4.07 × 10^− 5^	*ARHGAP10*, *NR3C2*
ARS-BFGL-NGS-11,930	17	18,316,220	[T/C]	0.087	4.95 × 10^− 5^	*MAML3*, *MGST2*, *LOC782412*, *SETD7*

SNP, single nucleotide polymorphism; BTA, *Bos taurus* autosome; bp, base pair; MAF, minor allele frequency. The reference genome assembly utilized for GWAS is Bos_taurus_UMD_3.1.1.

### Marker loci associated with GL

**Fig. 7:**
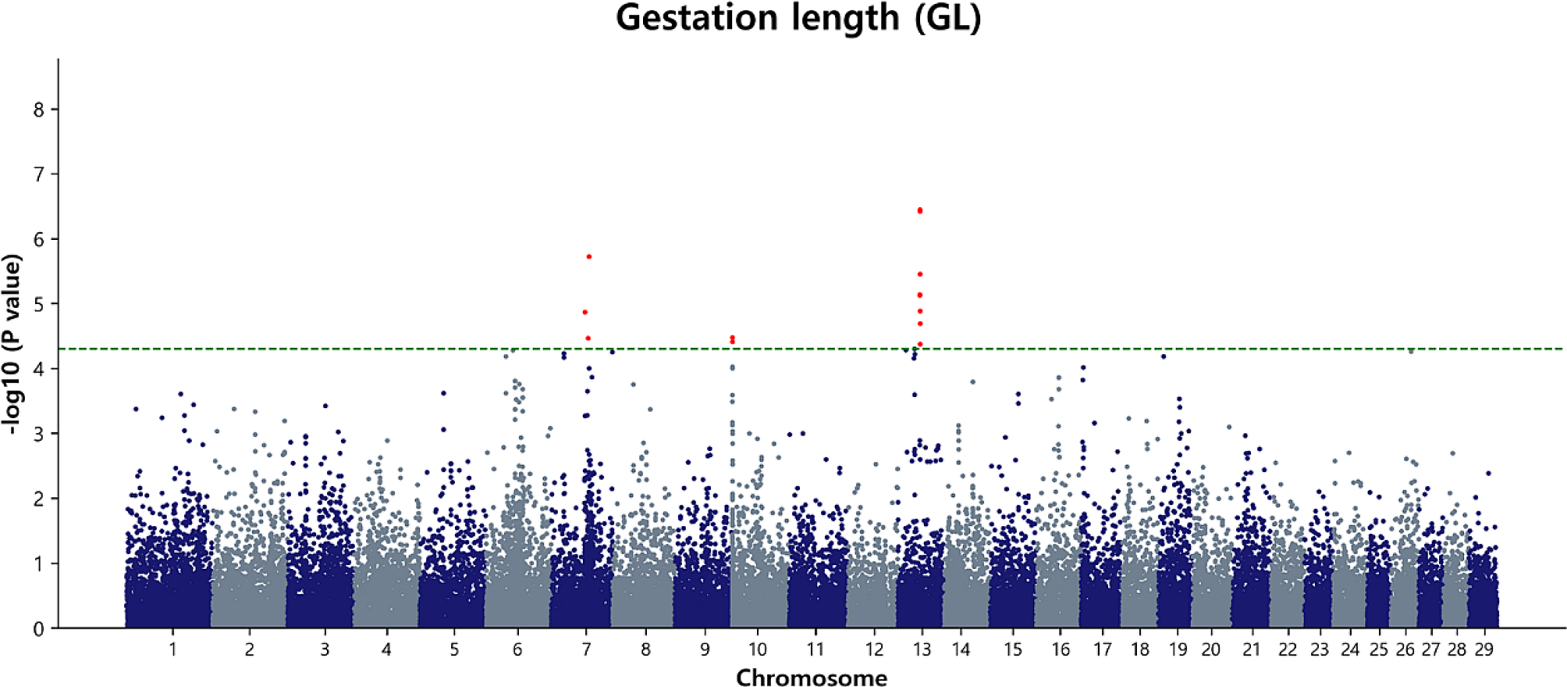
Manhattan plot of GWAS for GL in Hanwoo cows. The y-axis represents -log_10_ (observed) p-values for genome-wide SNPs against their respective positions on each chromosome (x-axis). The horizontal green line indicates the suggestive (5 × 10^− 5^) threshold level

The GWAS for GL in Hanwoo cattle revealed 13 significant SNPs (Table 4; Fig. 7). Notably, BTA13 exhibited the highest number of associated SNPs, totaling eight variants, suggesting a potential genomic hotspot for genetic factors influencing GL. Three SNPs were identified on BTA7, and these genetic markers were distributed across a genomic range, spanning positions from 62.02 Mb to 69.51 Mb. Additionally, BTA10 featured two SNPs associated with GL, positioned at 0.42 Mb and 0.43 Mb, respectively. Similarly, BTA13 emerged as a prominent genomic region with many identified SNPs related to GL, spanning positions from 40.78 Mb to 41.53 Mb. Among the top SNPs associated with GL, the five most significant SNPs were BTA-32,481-no-rs, *p* = 3.60 × 10^− 7^; ARS-BFGL-NGS-60,607, *p* = 3.80 × 10^− 7^; BovineHD0700020355, *p* = 1.90 × 10^− 6^; ARS-BFGL-NGS-113,658, *p* = 3.54 × 10^− 6^; and ARS-BFGL-NGS-37,354, *p* = 7.30 × 10^− 6^. Additional file 1: Fig S1 shows the regional association plot and LD heatmap for the BTA7, BTA10, and BTA13 regions. The QQ plot for the GL exhibited a normal distribution of p-values, with λ values (1.045) close to 1, indicating effective control of spurious results and a high likelihood of true associations (Additional file 2: Fig S2c).

**Table 4:** Genome-wide significant SNPs underlying GL in Hanwoo cows

SNP ID	BTA	Position (bp)	Allele	MAF	*p*-value	Gene
BTB-01700292	7	62,020,599	[A/C]	0.496	1.36 × 10^− 5^	*FBXO38*, *HTR4*
BovineHD0700019851	7	67,794,463	[C/T]	0.449	3.44 × 10^− 5^	*GALNT10*, *HAND1*, *SAP30L*, *LOC784642*, *LARP1*
BovineHD0700020355	7	69,509,097	[C/T]	0.250	1.90 × 10^− 6^	*SGCD*
BovineHD1000000074	10	415,234	[T/C]	0.265	3.36 × 10^− 5^	*LOC100295709*, *LOC100335191*, *LOC783211*, *LOC783248*, *LOC100297983*, *MCC*
BovineHD1000000082	10	427,698	[T/G]	0.265	3.90 × 10^− 5^	*LOC100295709*, *LOC100335191*, *LOC783211*, *LOC783248*, *LOC100297983*, *MCC*
BTA-32,481-no-rs	13	41,111,195	[C/T]	0.226	3.60 × 10^− 7^	*PLK1S1*, *LOC100140493*, *XRN2*, *LOC787396*, *NKX2-2*, *LOC614124*, *PAX1*
ARS-BFGL-NGS-60,607	13	41,164,722	[A/G]	0.226	3.80 × 10^− 7^	*XRN2*, *LOC787396*, *NKX2-2*, *LOC614124*, *PAX1*
ARS-BFGL-NGS-113,658	13	41,236,006	[G/A]	0.267	3.54 × 10^− 6^	*LOC787396*, *NKX2-2*, *LOC614124*, *PAX1*
ARS-BFGL-NGS-37,354	13	40,837,170	[C/T]	0.213	7.30 × 10^− 6^	*PLK1S1*, *LOC100140493*, *XRN2*, *LOC787396*
ARS-BFGL-NGS-41,919	13	40,777,908	[T/G]	0.208	7.51 × 10^− 6^	*PLK1S1*, *LOC100140493*, *XRN2*
ARS-BFGL-NGS-101,382	13	41,366,870	[A/G]	0.490	1.31 × 10^− 5^	*LOC132346904*, *FOXA2*
ARS-BFGL-NGS-102,025	13	41,529,941	[T/C]	0.412	2.04 × 10^− 5^	*FOXA2*
ARS-BFGL-NGS-115,482	13	41,337,809	[T/C]	0.336	4.23 × 10^− 5^	*PAX1*

SNP, single nucleotide polymorphism; BTA, *Bos taurus* autosome; bp, base pair; MAF, minor allele frequency. The reference genome assembly utilized for GWAS is Bos_taurus_UMD_3.1.1.

### Marker loci associated with NAIPC

**Fig. 8:**
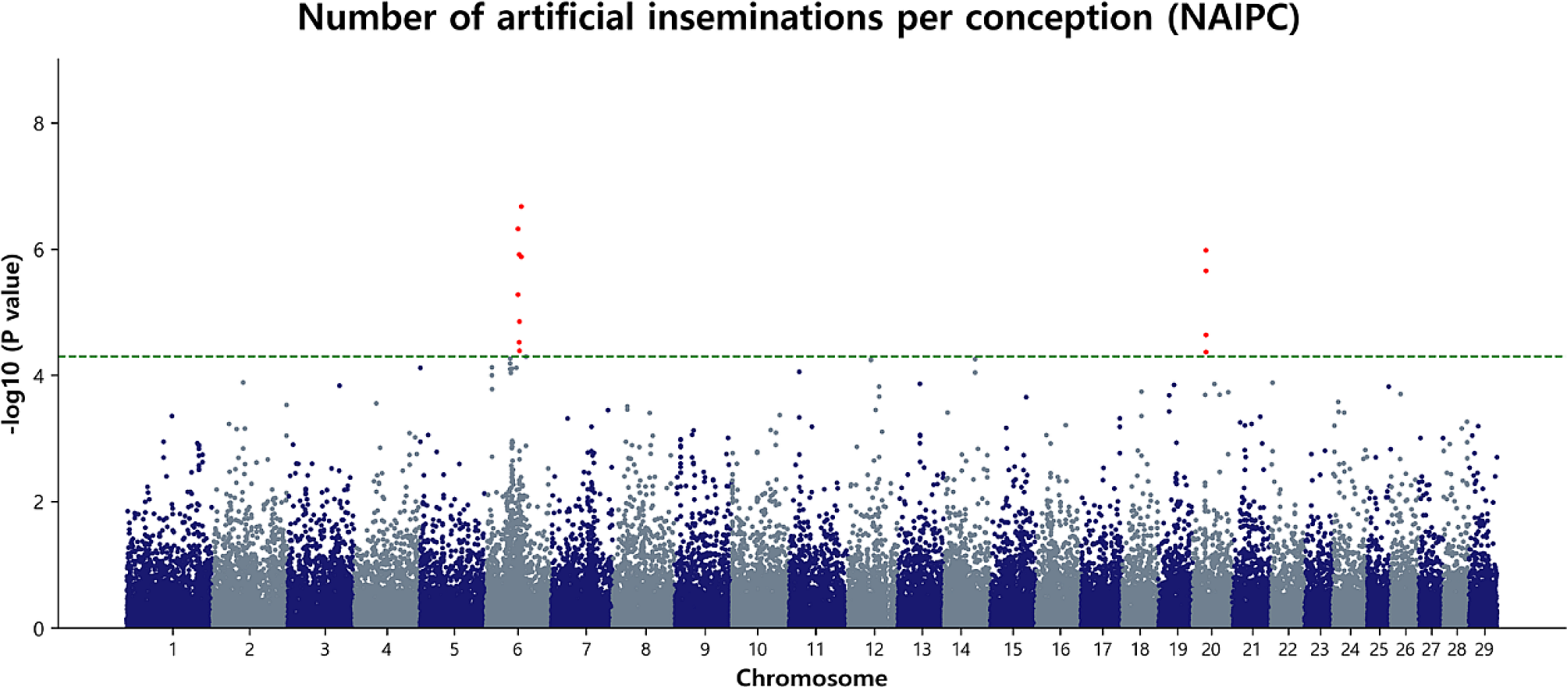
Manhattan plot of GWAS for NAIPC in Hanwoo cows. The y-axis represents -log_10_ (observed) p-values for genome-wide SNPs against their respective positions on each chromosome (x-axis). The horizontal green line indicates the suggestive (5 × 10^− 5^) threshold level

The GWAS for NAIPC in Hanwoo unveiled eight significant SNPs on BTA6, spanning from 58.39 Mb to 64.58 Mb. Simultaneously, BTA20 revealed four SNPs, covering regions from 23.72 Mb to 23.89 Mb (Table 5; Fig. 8). Notably, the most significant marker associated with NAIPC was identified on BTA6 at 64.58 Mb (BTB-01312166, *p* = 2.12 × 10^− 7^). Following closely, the second-most significant SNP was located on BTA6 at 58.41 Mb, influencing NAIPC in Hanwoo (BovineHD4100005053, *p* = 4.80 × 10^− 7^). These findings underscore the specific genetic variations associated with NAIPC on chromosomes 6 and 20 in Hanwoo cattle. The association plot and LD heat map for the BTA6 and BTA20 regions are depicted in Additional file 1: Fig S1. Furthermore, the QQ plot generated for NAIPC exhibited a normal distribution of p-values, with λ values (1.044) closely approximating 1 (Additional file 2: Fig S2d). This suggested an effective control of spurious results and a high probability of true associations, further validating the GWAS findings.

**Table 5:** Genome-wide significant SNPs underlying NAIPC in Hanwoo cows

SNP ID	BTA	Position (bp)	Allele	MAF	*p*-value	Gene
BovineHD0600016010	6	58,391,925	[A/G]	0.203	5.27 × 10^− 6^	*LOC100298905*, *LOC539460*, *LOC511424*, *LOC100336159*, *RELL1*
BovineHD4100005053	6	58,411,107	[A/C]	0.06516	4.80 × 10^− 7^	*LOC100298905*, *LOC539460*, *LOC511424*, *LOC100336159*, *RELL1*
BovineHD0600016641	6	60,526,521	[A/C]	0.4468	3.00 × 10^− 5^	*C6H4orf34*, *UBE2K*, *PDS5A*
BovineHD0600016649	6	60,559,199	[C/A]	0.4239	1.22 × 10^− 6^	*UBE2K*, *PDS5A*, *N4BP2*
BovineHD0600016807	6	61,137,644	[G/A]	0.4726	1.41 × 10^− 5^	*CHRNA9*, *RBM47*, *NSUN7*
BovineHD0600016826	6	61,197,209	[G/A]	0.3009	4.09 × 10^− 5^	*RBM47*, *NSUN7*, *APBB2*
Hapmap40687-BTA-21,304	6	64,542,255	[C/T]	0.1719	1.33 × 10^− 6^	*KCTD8*
BTB-01312166	6	64,578,869	[T/C]	0.07082	2.12 × 10^− 7^	*KCTD8*
Hapmap48670-BTA-16,740	20	23,717,482	[G/A]	0.4508	1.05 × 10^− 6^	*LOC100336745*, *LOC100337033*, *PPAP2A*, *SKIV2L2*, *DHX29*
BTA-88,011-no-rs	20	23,843,046	[C/T]	0.4477	2.21 × 10^− 6^	*PPAP2A*, *SKIV2L2*, *DHX29*, *LOC100301247*, *LOC100301340*
BTA-88,016-no-rs	20	23,864,571	[C/T]	0.4033	2.29 × 10^− 5^	*PPAP2A*, *SKIV2L2*, *DHX29*, *LOC100301247*, *LOC100301340*, *MIR449C*, *MIR449B*, *MIR449A*, *GPX8*, *CDC20B*, *GZMA*
BTA-88,001-no-rs	20	23,886,196	[T/G]	0.3762	4.28 × 10^− 6^	*PPAP2A*, *SKIV2L2*, *DHX29*, *LOC100301247*, *LOC100301340*, *MIR449C*, *MIR449B*, *MIR449A*, *GPX8*, *CDC20B*, *GZMA*

SNP, single nucleotide polymorphism; BTA, *Bos taurus* autosome; bp, base pair; MAF, minor allele frequency. The reference genome assembly utilized for GWAS is Bos_taurus_UMD_3.1.1.

### Identification of candidate genes and functional annotation

We explored the genes surrounding our reported significant marker loci associated with reproductive traits in Hanwoo by searching the National Center for Biotechnology Information (NCBI) database on cattle (*Bos taurus*) based on the Bos_taurus_UMD_3.1.1 genome assembly, considering a search window of ± 500 Kb around the identified marker loci. Considering the observed LD decay distance in the studied population, it is plausible that the causal mutations and genes were situated within a region spanning 500 kb, both upstream and downstream of the identified GWAS signals (Fig. 3c). A total of 138 unique positional candidate genes were identified within a 1 Mb region centered in proximity to the significant marker loci underlying reproductive traits in Hanwoo cattle (Tables 2, 3 and 4, and 5). Notably, we identified 37 nearby genes for AFC, 43 genes for CI, 23 genes for GL, and 27 genes for NAIPC.

**Fig. 9:**
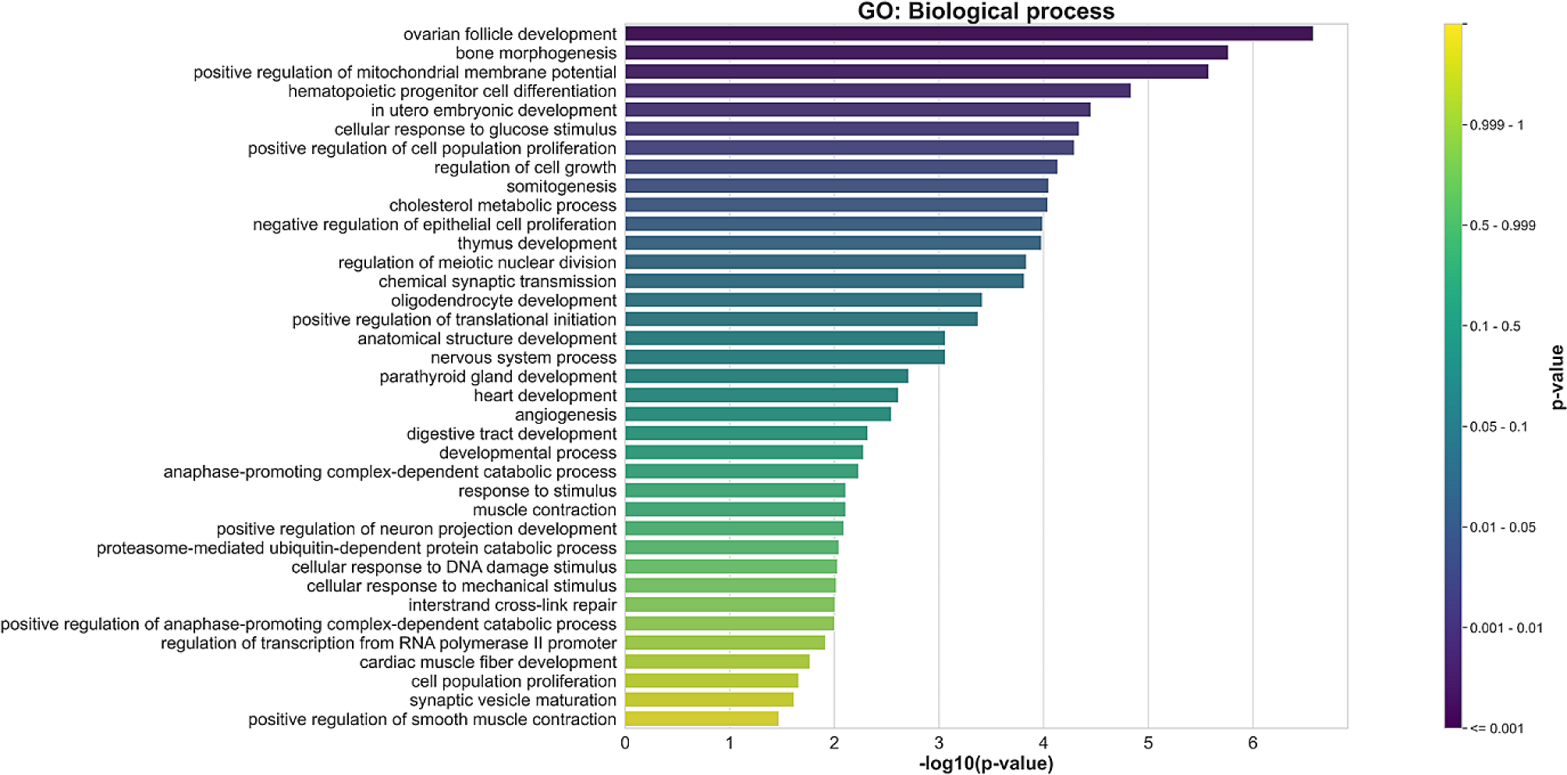
Bar plot illustrating the -log_10_ of the p-values for selected GO terms related to biological processes

Functional annotation of Gene Ontology (GO) terms was initially performed to identify the biological meaning and systematic features of the candidate genes using the Database for Annotation, Visualization, and Integrated Discovery (DAVID) and KEGG Orthology-Based Annotation System (KOBAS). The gene ontology encompassed four categories: Biological Process (BP), Molecular Functions (MF), and Cellular Component (CC), and Kyoto Encyclopedia of Genes and Genomes (KEGG). Candidate genes associated with reproductive traits exhibited significantly different GO terms (*P* < 0.05). Specifically, 37, 13, 11, and 8 GO terms were linked to BP, MF, CC, and KEGG, respectively (Fig. 9 and Additional file 3: Fig S3).

The pathway enrichment analysis revealed that genes involved in AFC, CI, GL, and NAIPC were enriched in chemical synaptic transmission (GO:0007268), regulation of transcription from RNA polymerase II promoter (GO:0006357), proteasome-mediated ubiquitin-dependent protein catabolic process (GO:0043161), cellular response to DNA damage stimulus (GO:0006974), interstrand cross-link repair (GO:0036297), positive regulation of neuron projection development (GO:0010976), cardiac muscle fiber development (GO:0048739), parathyroid gland development (GO:0060017), cell population proliferation (GO:0008283), cellular response to glucose stimulus (GO:0071333), developmental process (GO:0032502), bone morphogenesis (GO:0060349), ovarian follicle development (GO:0001541), regulation of cell growth (GO:0001558), anatomical structure development (GO:0048856), somitogenesis (GO:0001756), cellular response to mechanical stimulus (GO:0071260), thymus development (GO:0048538), hematopoietic progenitor cell differentiation (GO:0002244), heart development (GO:0007507), angiogenesis (GO:0001525), in utero embryonic development (GO:0001701), transcriptional activator activity (GO:0001228), transcription factor activity (GO:0003700), RNA polymerase II core promoter proximal region sequence-specific DNA binding (GO:0000978), glutathione peroxidase activity (GO:0004602), transcription cofactor binding (GO:0001221), RNA binding (GO:0003723), bHLH transcription factor binding (GO:0043425), RNA polymerase II transcription factor activity (GO:0000981), nucleus (GO:0005634), RNA polymerase II transcription factor complex (GO:0090575), Golgi membrane (GO:0000139), synapse (GO:0045202), neuronal cell body (GO:0043025), thyroid hormone synthesis (bta04918), metabolic pathways (bta01100), longevity regulating pathway (bta04211), relaxin signaling pathway (bta04926), and estrogen signaling pathway (bta04915). The functional gene set annotations and enrichment details are presented in Additional file 4: Table S1.

Subsequently, we used the GeneCards and Mouse Genome Informatics databases and conducted an extensive literature review to explore the functional roles of the identified genes. Positional candidate genes with functional biological roles related to reproductive traits, or those previously reported to be associated with reproductive traits, were regarded as promising candidates (Table 6). Consequently, we identified ten candidate genes with potential relevance to age at first calving. These genes, namely *FANCG*, *UNC13B*, *TESK1*, *TLN1*, *CREB3*, *FAM110B*, *UBXN2B*, *SDCBP*, *TOX*, and *MAP3K1* that were located on BTA8, BTA14, and BTA20, exhibited promising associations with AFC based on their known functions and previous research findings. Furthermore, *APBA3*, *TCF12*, and *ZFR2*, located on BTA7 and BTA10 were involved in the calving interval; *PAX1*, *SGCD*, and *HAND1*, located on BTA7 and BTA13, were associated with gestational length; and *RBM47*, *UBE2K*, and *GPX8* located on BTA6 and BTA20, were related to the number of artificial inseminations per conception in Hanwoo cows.

**Table 6:** Promising candidate genes associated with reproductive traits in Hanwoo cows

Traits	Genes	Name	BTA	Consequence type	QTL Position (Mb)
AFC	*FANCG*	Fanconi anemia complementation group G	8	DGV	59.75–59.76
AFC	*UNC13B*	Unc-13 homolog B	8	IV	59.76–60.03
AFC	*TESK1*	Testis associated actin remodelling kinase 1	8	UGV	60.21–60.21
AFC	*TLN1*	Talin 1	8	DGV	60.28–60.32
AFC	*CREB3*	CAMP responsive element binding protein 3	8	UGV	60.31–60.32
AFC	*FAM110B*	Family with sequence similarity 110 member B	14	IV	26.04–26.12
AFC	*UBXN2B*	UBX domain protein 2B	14	IV	26.27–26.31
AFC	*SDCBP*	Syndecan binding protein	14	IV	26.41–26.45
AFC	*TOX*	Thymocyte selection associated high mobility group box	14	IV	26.60–26.97
AFC	*MAP3K1*	Mitogen-activated protein kinase kinase kinase 1	20	DGV	22.35–22.44
CI	*ZFR2*	Zinc finger RNA binding protein 2	7	IV	21.35–21.41
CI	*APBA3*	Amyloid beta precursor protein binding family A member 3	7	UGV	21.44–21.45
CI	*TCF12*	Transcription factor 12	10	IV	53.05–53.52
GL	*HAND1*	Heart and neural crest derivatives expressed 1	7	IV	67.72–67.72
GL	*SGCD*	Sarcoglycan delta	7	IV	69.27–70.10
GL	*PAX1*	Paired box 1	13	IV	41.25–41.26
NAIPC	*UBE2K*	Ubiquitin conjugating enzyme E2 K	6	IV	60.41–60.50
NAIPC	*RBM47*	RNA binding motif protein 47	6	IV	61.01–61.34
NAIPC	*GPX8*	Glutathione peroxidase 8	20	DGV	23.98–23.98

BTA, *Bos taurus* autosomes; DGV, downstream gene variant; IV, intergenic variant; UGV, upstream gene variant.

## Discussion

Efficient reproductive traits are pivotal for the success of Hanwoo cattle farming and influence genetic improvement, economic viability, and overall herd productivity. The optimal performance of traits, such as age at first calving, calving interval, gestation length, and number of artificial inseminations per conception, plays a vital role in selective breeding programs, leading to the propagation of desirable genetic characteristics within the Hanwoo population. Their direct impact on productivity, rapid return on investment, and overall sustainability in the Hanwoo cattle industry underscores the economic significance of these traits [44]. Moreover, a keen understanding of reproductive traits will enable farmers to implement effective farm management practices, ensuring timely breeding and proper adaptation to environmental conditions [45].

### Candidate gene functions associated with AFC

By annotating gene functions, we identified five candidate genes associated with AFC on BTA8. These genes were *FANCG*, *UNC13B*, *TESK1*, *TLN1*, and *CREB3*. The *FANCG* (Fanconi anemia complementation group G) gene, positioned between 59.75 Mb and 59.76 Mb, encodes a protein expressed in the chromosomal instability syndrome associated with various developmental abnormalities, progressive bone marrow failure, reduced fertility, retarded growth, hyperpigmentation, and a predisposition to cancer [46, 47]. Fanconi anemia (FA) is a genetically heterogeneous, recessive disorder characterized by cytogenetic instability, hypersensitivity to DNA cross-linking agents, increased chromosomal breakage, and defective DNA repair. This gene encodes a protein for complementation group G (https://www.genecards.org/cgi-bin/carddisp.pl?gene=FANCG). As reported by Guitton-Sert et al. [47], analysis of embryos revealed germ cell aplasia occurring in the embryonic stages in the majority of FA mouse models. The earliest mammalian germ cells, known as primordial germ cells (PGCs), undergo extensive epigenetic reprogramming before progressing to meiosis. The onset of embryonic development relies on fundamental interactions between the sperm and oocytes [48]. Prior to this interaction, the sperm acrosome undergoes a series of transformations essential for its fusion with the zona pellucida. This intricate biological process, known as the acrosome reaction, involves acrosomal exocytosis, substructural remodeling, and various biochemical modifications [49]. These sequential processes are indispensable for successfully fusing sperm with the oocyte plasma membrane during a single fertilization event [50]. Positioned in the 59.76 Mb to 60.03 Mb region, the *UNC13B* (Unc-13 homolog B) gene plays a pivotal role in ensuring the normal functioning of the aforementioned mechanisms, ultimately contributing to fertility success [49]. The expression pattern of *UNC13B* closely aligns with these essential processes and is associated with immune response and cell differentiation. Consequently, *UNC13B* has emerged as a potential target for unraveling the intricate connection between immune response and fertility [49]. Testis-associated actin remodeling kinase 1 (*TESK1*) is a member of a conserved gene family known for its widespread functionality across various cellular processes, with particularly high expression in the testis [51]. According to Toshima et al. [52], *TESK1* is expressed in different tissues and cell lines, albeit at relatively low levels. This observation led to the assumption that *TESK1* might have general cellular functions rather than being testis-specific. Moreover, their research identified differential expression of the *TESK1* gene with reproductive and developmental processes. Numerous animal and human studies have associated this gene with fertility and infertility [53]. Notably, studies have confirmed the presence of *TESK1* in spermatozoa, suggesting its potential regulatory role during chromosomal repackaging in spermatozoa and post-fertilization in oocytes [54, 55]. Another important candidate gene associated with AFC is *TLN1* (Talin 1), located in the genomic region spanning from 60.28 Mb to 60.32 Mb. *TLN1* is important in various aspects of embryonic development, physiology, and disease processes. It performs various functions associated with integrins and directly influences diverse aspects of biology and medicine. These encompass critical stages in mammalian pregnancy, particularly implantation of the blastocyst onto the uterine wall, subsequent placentation, and conceptus development [56]. Studies on *TLN1* indicate that tamoxifen-induced inactivation of the talin1 gene across the embryo results in an angiogenesis phenotype confined to newly formed blood vessels. This phenotype manifests rapidly in early embryos, leading to vessel defects within 48 h and embryo death within 72 h [57]. *TLN1* is predominantly expressed in maternal epithelium and trophoblast giant cells (TGC). As pregnancy progresses, *TLN1* expression increases in the TGC and the surrounding caruncular epithelial cells while it diminishes in the basal compartment of the caruncular epithelial cells in bovines [58]. This dynamic expression pattern underscores *TLN1*’s integral involvement at various pregnancy stages. An additional noteworthy candidate gene associated with AFC is *CREB3* (CAMP responsive element binding protein 3), situated on BTA8 within the genomic region spanning from 60.31 Mb to 60.32 Mb. This gene is significant in the decidualization of endometrial stromal cells (ESCs) [59]. Additionally, elevated expression levels of *CREB3* were observed in decidualized cells on days 6–8 of pregnancy, highlighting the potential regulatory influence of *CREB3* on the decidualization pathway [60, 61].

Furthermore, we identified four candidate genes associated with AFC on BTA14, namely *FAM110B*, *UBXN2B*, *SDCBP*, and *TOX*, along with one gene on BTA20, identified as *MAP3K1*. The positional candidate gene identified in our study, located around the significant SNP on BTA14 spanning from 26.04 Mb to 26.97 Mb and associated with AFC in Hanwoo cattle, aligns with previous research. Specifically, a study on Nellore cattle reported an association of the genes *UBXN2B* (UBX domain protein 2B) and *SDCBP* (Syndecan binding protein) with AFC [62]. Similarly, in Brahman cattle, the *FAM110B* (Family with sequence similarity 110 member B) and *TOX* (Thymocyte selection associated high mobility group box) genes were linked to puberty-related traits, including age at the formation of the first corpus luteum [63]. Notably, *FAM110B*, *UBXN2B*, and *TOX*, located in the genomic region of 26.04–26.97 Mb on chromosome 14, have previously been shown to be associated with various traits such as growth, birth weight, carcass weight, average daily gain, feed intake, meat tenderness, height, and stature across different beef cattle breeds [64–66]. Additionally, the *TOX* gene, previously identified in genome-wide association studies on Nellore females, has been linked to reproductive traits [67]. Furthermore, *SDCBP*, mapped to a conserved region on chromosome 14, has emerged as a candidate gene for determining carcass weight in Hanwoo cattle, given its role in binding to various transmembrane proteins [68–70].

The gene *MAP3K1* (Mitogen-activated protein kinase kinase kinase 1), located on BTA20 spanning from 22.35 Mb to 22.44 Mb, is a key regulator of MAPK activation. This activation is integral to various cellular processes, including gene expression, cell proliferation, migration, survival, and death [71]. Moreover, extensive studies in the human genomic context have revealed a robust correlation between *MAP3K1* mutations and sexual development and differentiation [72, 73]. *MAP3K1* functions as a dynamic signaling molecule with diverse cell type-specific roles and contributes to the development of the female reproductive tract. Notably, females deficient in the kinase domain of *MAP3K1* present with conditions such as imperforate vagina, labor failure, and infertility, highlighting the indispensable role of *MAP3K1* in reproductive health [74].

### Candidate gene functions associated with CI

The CI in Hanwoo cattle, which denotes the time lapse between successive calving events in an individual cow, is a major reproductive trait with multifaceted implications. It indicates reproductive efficiency by influencing the frequency and yield of offspring within a defined timeframe. Additionally, the calving interval is important in herd management strategies, enabling farmers to strategically plan breeding cycles and allocate resources [75]. Economic ramifications are substantial, as a shorter calving interval enhances turnover rates and contributes to the financial viability of Hanwoo cattle farming [76]. In this study, we identified the presence of the *ZFR2* and *APBA3* genes on BTA7, whereas the *TCF12* gene was identified on BTA10 near the significant SNPs associated with CI in Hanwoo cows. Specifically, the zinc finger RNA binding protein 2 (*ZFR2*) was located within the genomic range of 21.35 Mb to 21.41 Mb. Notably, mutations in *ZFR2* have been implicated as a potential cause of primary ovarian insufficiency in women, demonstrating an association with a complete lack of oocytes and follicles [77]. Additionally, the amyloid beta precursor protein binding family A member 3 (*APBA3*) gene, situated in the region from 21.44 Mb to 21.45 Mb, has been linked to ovarian hyperstimulation for in vitro fertilization in women [78]. Furthermore, the transcription factor 12 (*TCF12*) gene, found between 53.05 Mb and 53.52 Mb, has been observed to play a role in muscle development and regeneration, as indicated by loss-of-function studies, and is important in regulating cell growth and differentiation during embryonic development [79].

### Candidate gene functions associated with GL

We have identified two noteworthy candidate genes, *HAND1* and *SGCD*, associated with gestation length on BTA7 spanning from 67.72 Mb to 70.10 Mb, and *PAX1* on BTA13 ranging from 41.25 Mb to 41.26 Mb. The *HAND1* gene, which encodes heart and neural crest derivatives expressed 1, is linked to congenital heart disease (CHD) and is expressed in placental trophoblasts and endothelial cells in various mouse models [80]. Histological examination of the placenta revealed that the loss of *HAND1* in labyrinthine progenitor trophoblasts during early pregnancy significantly affected syncytial layer formation and labyrinthine vasculature development [80]. *HAND1*, a basic helix-loop-helix transcription factor, is involved in multiple organ systems during embryogenesis. *SGCD* gene sarcoglycan delta may contribute to the embryonic heart response in animals and is associated with autosomal recessive limb-girdle muscular dystrophy and dilated cardiomyopathy due to *SGCD* mutations [81]. Additionally, the *PAX1* gene, a paired box 1 transcription factor, is important for the development of various tissues during embryogenesis [82], including the thymus [83, 84], vertebral column [85], chondrogenic differentiation, and chondrocyte maturation [86]. These findings shed light on the roles of *HAND1*, *SGCD*, and *PAX1* in regulating gestation length and their broader implications in embryonic development.

### Candidate gene functions associated with NAIPC

The NAIPC is a metric used in animal reproduction, particularly in livestock breeding. It quantifies the average number of artificial insemination attempts required for successful conception or pregnancy. This metric is valuable for assessing the efficiency of artificial insemination techniques, reproductive performance, and overall breeding management strategies. A lower number indicates higher reproductive efficiency, whereas a higher number suggests challenges or inefficiencies in the artificial insemination process [87]. Monitoring and optimizing this parameter in Hanwoo cattle breeding programs are essential for optimizing reproductive outcomes, refining breeding strategies, ensuring overall health, and maximizing reproductive success in Hanwoo populations. We identified three promising candidate genes associated with NAIPC, BTA6, and BTA20 in Hanwoo cattle through gene function annotation. The *UBE2K* (Ubiquitin-conjugating enzyme E2 K) and *RBM47* (RNA binding motif protein 47) genes are situated on BTA6 between 60.41 Mb and 61.34 Mb, whereas *GPX8* (glutathione peroxidase 8) is located on BTA20 at 23.98 Mb. *UBE2K* is associated with reproductive failure, specifically during gametogenesis and embryogenesis [88]. Similarly, *RBM47* influences mouse blastocyst development, with transcriptional expression observed during the preimplantation stages. Immunofluorescence analysis indicated that the *RBM47* protein was first detected in morula-stage embryos and was primarily localized in the nucleus of blastocyst embryos [89]. Previous studies by Shivalingappa et al. [90] suggested multifunctional roles of *RBM47* in processes such as RNA editing and transcriptional activation during blastocyst development. In a rat experiment by Mihalik et al. [91], *GPX8* was observed throughout the preimplantation period from unfertilized oocytes to blastocysts. Notably, *GPX8* was detected in the ovary, uterine tube, and uterus of the mother, with the highest protein levels observed on day one of pregnancy, which gradually declined thereafter. Immunohistochemistry revealed *GPX8* in Graafian follicles within the ovary, and immunofluorescence confirmed its presence in ovulated oocytes and corona radiata cells of the oviduct. These findings highlight the potential roles of *UBE2K*, *RBM47*, and *GPX8* in the Hanwoo cow reproductive processes.

## Conclusion

GWAS studies with a large sample size are a potential tool for uncovering novel genomic regions associated with traits of interest. It is important to note that multiple genes likely influence reproductive traits, each contributing a small proportion to the observed variation. In addition, mapping genes to SNP loci may not always lead to identifying genes within or near the identified SNPs. Since there is no prior report of GWAS for reproductive traits in Korean Hanwoo cattle, the current study’s findings serve as a foundational baseline, requiring further research for validation. Concerns regarding the effects of breed composition and population structure on GWAS results should be acknowledged. In the present GWAS for key reproductive traits in Korean Hanwoo cattle, we successfully identified chromosomal regions that contribute to a deeper understanding of the genetic and physiological mechanisms regulating these traits, along with candidate genes for investigating causal mutations. In total, 68 significant genome-wide SNPs were detected, with BTA6, BTA7, BTA8, BTA10, BTA13, BTA14, BTA17, and BTA20 emerging as the prominent genomic regions. Through gene function annotation, we identified *FANCG*, *UNC13B*, *TESK1*, *TLN1*, *CREB3*, *FAM110B*, *UBXN2B*, *SDCBP*, *TOX*, *MAP3K1*, *APBA3*, *TCF12*, *ZFR2*, *PAX1*, *SGCD*, *HAND1*, *RBM47*, *UBE2K*, and *GPX8* as the most promising candidate genes for age at first calving, calving interval, gestation length, and the number of artificial inseminations per conception, respectively. These findings hold significant promise for future marker-assisted selection in Hanwoo cattle breeding programs and provide a pathway for enhanced trait selection and genetic improvement of this valuable breed. In conclusion, our groundbreaking GWAS analysis illuminates a path toward unraveling the genetic intricacies of Korean Hanwoo cattle reproductive traits, opening avenues for innovative advancements in cattle breeding practices.

## Materials and methods

### Animals and phenotypes

First-parity data were collected from 11,348 Hanwoo cows from nine commercial herds in the South Korean province of Gyeongsanbuk-do. Four female reproductive traits, namely AFC, CI, GL, and NAIPC, were examined. The AFC, CI, and GL measurements were recorded in days, whereas NAIPC was recorded as the total number of occurrences.

### Genotyping and quality control

A total of 11,348 Hanwoo cows were genotyped using an Illumina Bovine 50 K SNP chip (Illumina Inc., San Diego, CA, USA), where 53,866 SNPs were embedded. We excluded SNPs located in duplicate sex chromosomes or in uncertain positions to ensure data quality, resulting in the removal of 1750 SNPs. This process yielded 52,116 SNPs for subsequent analysis. For further refinement, several quality control (QC) criteria were applied to filter out low-quality SNPs. Specifically, SNPs with a minor allele frequency (MAF) below 5% (i.e., monomorphic; 9281 SNPs), an SNP call rate below 90% (732 SNPs), individuals with a genotyping call rate less than 90% (*N* = 62), and SNPs showing a significant deviation from Hardy–Weinberg equilibrium (HWE) with a p-value exceeding 10^− 6^ (1296 SNPs) were excluded from the dataset. An identity-by-state (IBS) test was conducted to identify genetically similar individuals or potential genotyping errors. Pairs of individuals with a similarity rate exceeding 99% were considered identical or indicative of genotyping errors (*N* = 48). The IBS and QC processes were performed using the PLINK v1.9 toolset [92]. Additionally, missing alleles were imputed using Beagle v5.4 software [93]. Following the IBS and QC procedures, 11,238 animals with genotypes of 40,807 SNPs remained available for subsequent analysis.

### Estimation of population structure and linkage disequilibrium

Principal component analysis (PCA) and linkage disequilibrium (LD) analyses were conducted on quality-controlled SNPs to explore the population structure of Hanwoo cattle. PCA was performed using the PLINK toolset v1.9 [92]. The average LD decay distance across the entire imputed genome of the Hanwoo population was calculated using TASSEL software v5.2.92 [94]. LD decay was visualized by plotting the distance against the average r^2^ value using the R package ggplot2 [95].

### GWAS analysis

Reproductive traits were analyzed using the linear mixed model (LMM) implemented in the genome-wide efficient mixed-model analysis (GEMMA) software v0.98.5 [96]. GEMMA computes a genomic relationship matrix (GRM) between individuals within each population to determine the population structure. The univariate linear mixed model in the GEMMA is described as follows:

$$\text{y}=\text{W}{\upalpha }+\text{X}{\upbeta }+\text{u}+{\upepsilon }$$

where y is the vector of phenotypes; W is the incidence matrix covariates, including fixed effects of the herd in which the animal was raised and the year and season of birth and calving; α is a vector of the corresponding coefficients, including the intercept; X represents the vector of all marker genotypes; β represents the effect size of the SNP; $\text{u} \sim \text{M}\text{V}{\text{N}}_{\text{n}}(0, {\uplambda }{{\uptau }}^{-1}\text{K})$ is an n-vector of animal additive effects; and ${\upepsilon } \sim \text{M}\text{V}{\text{N}}_{\text{n}}(0, {{\uptau }}^{-1}{\text{I}}_{\text{n}})$ represents an n-vector of errors; ${{\uptau }}^{-1}$ is the variance of the residual errors; λ is the ratio between the two variance components; K represents the genomic relationship matrix (GRM); ${\text{I}}_{\text{n}}$ is an n $\times$ n identity matrix; and $\text{M}\text{V}{\text{N}}_{\text{n}}$ represents the n-dimensional multivariate normal distribution. GEMMA calculates the GRM as follows [96]:

$$\text{G}=\frac{1}{\text{p}}\sum _{\text{i}=1}^{\text{p}}{({\text{x}}_{\text{i}}-{1}_{\text{n}}{\stackrel{-}{\text{x}}}_{\text{i}})({\text{x}}_{\text{i}}-{1}_{\text{n}}{\stackrel{-}{\text{x}}}_{\text{i}})}^{\text{T}}$$

where X represents the n × p matrix of the genotypes, ${\text{x}}_{\text{i}}$ represents the genotypes of the i^th^ SNP, ${\stackrel{-}{\text{x}}}_{\text{i}}$ is the sample mean, and ${1}_{\text{n}}$ is the *n* × 1 vector of 1.

Manhattan plots were generated to visualize the genome-wide distribution of significant SNPs. The significance level is represented as the negative base − 10 logarithm (-log_10_) of the p-value for each SNP. The Bonferroni test determined the genome-wide significance threshold (0.05/N, where N represents the number of SNPs). A more lenient threshold of 5 × 10^− 5^ (4.30) was implemented to identify suggestive SNPs, as suggested by the Wellcome Trust Case Control Consortium (https://www.wtccc.org.uk/), after recognizing the strictness of this criterion. The genomic inflation factor, lambda (λ), was calculated to assess population stratification by comparing the median chi-squared test statistics from GWAS to the expected median of the chi-squared distribution. In our study, p-values from GWAS results for all traits were used to compute λ using the qchisq() function in R [97]. Ideally, the genomic inflation factor should be close to 1 after adjusting for population stratification [39]. However, the notably high value of the genomic inflation factor suggests that factors beyond population stratification, such as strong linkage disequilibrium, significant associations between phenotypic traits and SNPs, or systematic technical biases, may contribute to the observed inflation [40]. Additionally, QQ plots were drawn to depict observed versus expected p-values (-log_10_P) for each trait.

### Analysis of haplotype block

GWAS often reveal significant SNPs associated with target traits in putative regions. Clustering of these SNPs may be attributed to high LD and non-random association of the alleles on the chromosome. We used PLINK v1.9 [92] and LDBlockShop software [98] for chromosomal regions in which multiple SNPs were significantly clustered around the top SNP to investigate these genomic patterns. This approach enabled us to examine LD patterns within these regions meticulously.

### Identification of candidate genes and analysis of functional enrichment

Putative candidate genes within the QTL regions, as well as in the nearest upstream and downstream regions (500 kb) of our mapped significant SNPs, were identified based on the bovine genome assembly (Bos_taurus_UMD_3.1.1) [22]. We used online resources, including Genome Data Viewer (https://www.ncbi.nlm.nih.gov/genome/gdv/org=bos-taurus; accessed on November 15, 2023), BovineMine v1.6 (an integrated data warehouse for the Bovine Genome Database (http://128.206.116.13:8080/bovinemine/begin.do, accessed on November 15, 2023), and BGVD (Bovine Genome Variation Database and Selective Signatures, available at http://animal.nwsuaf.edu.cn/code/index.php/BosVar, accessed on November 17, 2023). We conducted KEGG and GO analyses using DAVID [99] and KOBAS v3.0 [100] to investigate the functions of all candidate genes. Enriched terms with a significance threshold of *p*-value < 0.05 were selected to further explore the genes involved in pathways and biological processes. The functional roles of the identified genes within and near significant SNPs associated with reproductive traits were explored using published reports in PMC for Biotechnology Information (NCBI database) journals and other literature surveys. The functional roles of each gene were also obtained from online resources, including human gene functions at GeneCards (www.genecards.org), the Mouse Genome Informatics (MGI) website (https://www.informatics.jax.org/), and Ensembl (www.ensembl.org/biomart/martview), accessed on November 18, 2023. Candidate genes that appeared to be functionally related to the desired traits of interest were considered promising candidate genes.

### Electronic supplementary material

Below is the link to the electronic supplementary material.

**Additional file 1: Fig S1.** Regional association plot showing the distribution of significant loci associated with AFC, CI, GL, and NAIPC at various BTA (top), and heatmap of LD (bottom). Description: The red horizontal line indicates -log10P = 4.30

**Additional file 2: Fig S2** The quantile-quantile (QQ) plots and genomic inflation factor (λ) of the GWAS analysis for reproductive traits in Korean Hanwoo cows. Description: QQ plots showing late separation between the observed and expected p-values (-log10P). The genomic inflation factor (λ) is close to 1, indicating that there is no population stratification

**Additional file 3: Fig S3**. Bar plot of -log10 of the p-values of selected GO terms. Description: This file provides the GO term (*p* < 0.05) plot of molecular functions, cellular components, and KEGG pathways

**Additional file 4: Table S1**. GO terms were significantly enriched using candidate genes associated with the reproductive traits of Hanwoo cows. Description: This file provides GO terms (*P* < 0.05) enriched for candidate genes associated with reproductive traits

## Data Availability

The original genotypic data (SNPs) used in this study are accessible on Dryad (https://doi.org/10.5061/dryad.8sf7m0cxr). Access to the data for reproducing the results requires an application to, and permission from the authors. The phenotypic data analyzed in this study are not currently publicly available, as additional analytical studies will be conducted in the future. It can be obtained from the corresponding author upon reasonable request.

## Abbreviations

AFC: Age at first calving
BGVD: Bovine Genome Variation Database
BTA: *Bos taurus* autosomes
CI: Calving interval
DAVID: Database for Annotation, Visualization, and Integrated Discovery
GEMMA: Genome-wide Efficient Mixed Model Analysis
GL: Gestation length
GO: Gene ontology
GRM: Genomic relationship matrix
GWAS: Genome-wide association study
IBS: Identity-by-state
KEGG: Kyoto Encyclopedia of Genes and Genomes
LD: Linkage disequilibrium
LMM: Linear mixed model
PCA: Principal component analysis
QC: Quality control
QTL: Quantitative trait locus
NAIPC: Number of artificial inseminations per conception
NCBI: National Center for Biotechnology Information
SNP: Single nucleotide polymorphism

## References

[1] Kim S, Choi H, Alam J, Park M. MN: Breeding initiatives for Hanwoo cattle to thrive as a beef industry – A review study. J Anim Breed Genomics 2017, 1(2).

[2] Lopez BI, Son JH, Seo K, Lim D (2019). Estimation of genetic parameters for Reproductive traits in Hanwoo (Korean Cattle). Anim (Basel).

[3] Cavani L, Garcia DA, Carreno LO, Ono RK, Pires MP, Farah MM, Ventura HT, Millen DD, Fonseca R (2015). Estimates of genetic parameters for reproductive traits in Brahman cattle breed. J Anim Sci.

[4] Haque MA, Iqbal A, Alam M, Lee Y-M, Ha J-J, Kim J-J. Estimation of genetic correlations and genomic prediction accuracy for reproductive and carcass traits in Hanwoo cows. J Anim Sci Technol 2023. 10.5187/jast.2023.e75 PMC11331368 39165742

[5] MacNeil MD, Geary TW, Perry GA, Roberts AJ, Alexander LJ (2006). Genetic partitioning of variation in ovulatory follicle size and probability of pregnancy in beef cattle. J Anim Sci.

[6] Atashi H, Asaadi A, Hostens M (2021). Association between age at first calving and lactation performance, lactation curve, calving interval, calf birth weight, and dystocia in Holstein dairy cows. PLoS ONE.

[7] Brzakova M, Citek J, Svitakova A, Vesela Z, Vostry L (2020). Genetic parameters for age at First Calving and First Calving interval of beef cattle. Anim (Basel).

[8] Lopez-Paredes J, Perez-Cabal MA, Jimenez-Montero JA, Alenda R (2018). Influence of age at first calving in a continuous calving season on productive, functional, and economic performance in a blonde d’Aquitaine beef population. J Anim Sci.

[9] Morales R, Phocas F, Solé M, Demyda-Peyrás S, Menéndez-Buxadera A, Molina A (2017). Breeding beef cattle for an extended productive life: evaluation of selection criteria in the Retinta breed. Livest Sci.

[10] Fathoni A, Boonkum W, Chankitisakul V, Duangjinda M. An Appropriate Genetic Approach for Improving Reproductive Traits in crossbred thai-holstein cattle under heat stress conditions. Vet Sci 2022, 9(4). 10.3390/vetsci9040163 PMC9031002 35448661

[11] Do C, Wasana N, Cho K, Choi Y, Choi T, Park B, Lee D (2013). The effect of age at first calving and calving interval on productive life and lifetime profit in Korean holsteins. Asian-Australas J Anim Sci.

[12] Crowe MA, Hostens M, Opsomer G (2018). Reproductive management in dairy cows - the future. Ir Vet J.

[13] Pregnant cows. timing of pregnancy, open cows, pregnancy rate [https://beef.unl.edu/faq/pregnant-cows]].

[14] Piedrafita J, de la Torre JLR, Quintanilla R, Manteca X (2000). Variation in gestation length as breeding season advances in beef cattle breed. Ann Zootech.

[15] Norman HD, Wright JR, Kuhn MT, Hubbard SM, Cole JB, VanRaden PM (2009). Genetic and environmental factors that affect gestation length in dairy cattle. J Dairy Sci.

[16] Souames S, Berrama Z (2020). Factors affecting conception rate after the first artificial insemination in a private dairy cattle farm in North Algeria. Vet World.

[17] Chang YM, Andersen-Ranberg IM, Heringstad B, Gianola D, Klemetsdal G (2006). Bivariate analysis of number of services to conception and days open in Norwegian red using a censored threshold-linear model. J Dairy Sci.

[18] Tadesse B, Reda AA, Kassaw NT, Tadeg W (2022). Success rate of artificial insemination, reproductive performance and economic impact of failure of first service insemination: a retrospective study. BMC Vet Res.

[19] Wang X, Zhang Y, Sun HL, Wang LT, Li XF, Wang F, Wang YL, Li QC (2021). Factors affecting Artificial insemination pregnancy outcome. Int J Gen Med.

[20] Cardoso Consentini CE, Wiltbank MC, Sartori R (2021). Factors that Optimize Reproductive efficiency in dairy herds with an emphasis on timed Artificial Insemination Programs. Anim (Basel).

[21] Giordano JO, Sitko EM, Rial C, Perez MM, Granados GE. Symposium review: Use of multiple biological, management, and performance data for the design of targeted reproductive management strategies for dairy cows. *J Dairy Sci* 2022, 105(5):4669–4678. 10.3168/jds.2021-21476 35307173

[22] Haque MA, Alam MZ, Iqbal A, Lee YM, Dang CG, Kim JJ (2023). Genome-wide Association studies for body conformation traits in Korean Holstein Population. Anim (Basel).

[23] Liu A, Wang Y, Sahana G, Zhang Q, Liu L, Lund MS, Su G (2017). Genome-wide Association Studies for Female Fertility Traits in Chinese and nordic holsteins. Sci Rep.

[24] Stegemiller MR, Murdoch GK, Rowan TN, Davenport KM, Becker GM, Hall JB, Murdoch BM. Genome-Wide Association Analyses of Fertility Traits in Beef heifers. Genes (Basel) 2021, 12(2). 10.3390/genes12020217 PMC7913221 33540904

[25] Nayeri S, Sargolzaei M, Abo-Ismail MK, May N, Miller SP, Schenkel F, Moore SS, Stothard P (2016). Genome-wide association for milk production and female fertility traits in Canadian dairy Holstein cattle. BMC Genet.

[26] Berry DP, Bastiaansen JW, Veerkamp RF, Wijga S, Wall E, Berglund B, Calus MP (2012). Genome-wide associations for fertility traits in Holstein-Friesian dairy cows using data from experimental research herds in four European countries. Animal.

[27] Hyeong KE, Iqbal A, Kim JJ (2014). A genome wide Association Study on Age at First Calving using high density single nucleotide polymorphism chips in Hanwoo (Bos taurus coreanae). Asian-Australas J Anim Sci.

[28] Prakapenka D, Liang Z, Da Y. Genome-wide Association Study of Age at First Calving in U.S. Holstein cows. Int J Mol Sci 2023, 24(8). 10.3390/ijms24087109 PMC10138929 37108271

[29] Sanchez MP, Tribout T, Kadri NK, Chitneedi PK, Maak S, Hoze C, Boussaha M, Croiseau P, Philippe R, Spengeler M (2023). Sequence-based GWAS meta-analyses for beef production traits. Genet Sel Evol.

[30] Keogh K, Carthy TR, McClure MC, Waters SM, Kenny DA (2021). Genome-wide association study of economically important traits in Charolais and Limousin beef cows. Animal.

[31] Olsen HG, Hayes BJ, Kent MP, Nome T, Svendsen M, Larsgard AG, Lien S (2011). Genome-wide association mapping in Norwegian red cattle identifies quantitative trait loci for fertility and milk production on BTA12. Anim Genet.

[32] Kolbehdari D, Wang Z, Grant JR, Murdoch B, Prasad A, Xiu Z, Marques E, Stothard P, Moore SS (2009). A whole genome scan to map QTL for milk production traits and somatic cell score in Canadian holstein bulls. J Anim Breed Genet.

[33] Ilie DE, Mizeranschi AE, Mihali CV, Neamt RI, Goilean GV, Georgescu OI, Zaharie D, Carabas M, Hutu I. Genome-Wide Association Studies for milk somatic cell score in Romanian dairy cattle. Genes (Basel) 2021, 12(10). 10.3390/genes12101495 PMC8535694 34680890

[34] Wang P, Li X, Zhu Y, Wei J, Zhang C, Kong Q, Nie X, Zhang Q, Wang Z (2022). Genome-wide association analysis of milk production, somatic cell score, and body conformation traits in Holstein cows. Front Veterinary Sci.

[35] Buaban S, Lengnudum K, Boonkum W, Phakdeedindan P (2022). Genome-wide association study on milk production and somatic cell score for Thai dairy cattle using weighted single-step approach with random regression test-day model. J Dairy Sci.

[36] Zanella R, Settles ML, McKay SD, Schnabel R, Taylor J, Whitlock RH, Schukken Y, Van Kessel JS, Smith JM, Neibergs HL (2011). Identification of loci associated with tolerance to Johne’s disease in Holstein cattle. Anim Genet.

[37] Pant SD, Schenkel FS, Verschoor CP, You Q, Kelton DF, Moore SS, Karrow NA (2010). A principal component regression based genome wide analysis approach reveals the presence of a novel QTL on BTA7 for MAP resistance in holstein cattle. Genomics.

[38] Alonso-Hearn M, Badia-Bringue G, Canive M (2022). Genome-wide association studies for the identification of cattle susceptible and resilient to paratuberculosis. Front Veterinary Sci.

[39] Narayana SG, de Jong E, Schenkel FS, Fonseca PAS, Chud TCS, Powell D, Wachoski-Dark G, Ronksley PE, Miglior F, Orsel K (2023). Underlying genetic architecture of resistance to mastitis in dairy cattle: a systematic review and gene prioritization analysis of genome-wide association studies. J Dairy Sci.

[40] Zhou J, Liu L, Chen CJ, Zhang M, Lu X, Zhang Z, Huang X, Shi Y (2019). Genome-wide association study of milk and reproductive traits in dual-purpose Xinjiang Brown cattle. BMC Genomics.

[41] Dubon MAC, Pedrosa VB, Feitosa FLB, Costa RB, de Camargo GMF, Silva MR, Pinto LFB (2021). Identification of novel candidate genes for age at first calving in Nellore cows using a SNP chip specifically developed for Bos taurus indicus cattle. Theriogenology.

[42] Haque MA, Lee Y-M, Ha J-J, Jin S, Park B, Kim N-Y, Won J-I, Kim J-J (2024). Genomic predictions in Korean Hanwoo cows: a comparative analysis of genomic BLUP and bayesian methods for Reproductive traits. Animals-Basel.

[43] Singh A, Kumar A, Mehrotra A, A K, Pandey AK, Mishra BP, Dutt T (2021). Estimation of linkage disequilibrium levels and allele frequency distribution in crossbred Vrindavani cattle using 50K SNP data. PLoS ONE.

[44] Carvalho FE, Ferraz JBS, Pedrosa VB, Matos EC, Eler JP, Silva MR, Guimaraes JD, Bussiman FO, Silva BCA, Cancado FA (2023). Genetic parameters for various semen production and quality traits and indicators of male and female reproductive performance in Nellore cattle. BMC Genomics.

[45] Clarkson G, Dorward P, Poskitt S, Stern RD, Nyirongo D, Fara K, Gathenya JM, Staub CG, Trotman A, Nsengiyumva G (2022). Stimulating small-scale farmer innovation and adaptation with Participatory Integrated Climate Services for Agriculture (PICSA): lessons from successful implementation in Africa, Latin America, the Caribbean and South Asia. Clim Serv.

[46] Koomen M, Cheng NC, van de Vrugt HJ, Godthelp BC, van der Valk MA, Oostra AB, Zdzienicka MZ, Joenje H, Arwert F (2002). Reduced fertility and hypersensitivity to mitomycin C characterize Fancg/Xrcc9 null mice. Hum Mol Genet.

[47] Guitton-Sert L, Gao Y, Masson JY (2021). Animal models of fanconi anemia: a developmental and therapeutic perspective on a multifaceted disease. Semin Cell Dev Biol.

[48] Evans JP (2012). Sperm-egg interaction. Annu Rev Physiol.

[49] Pang WK, Amjad S, Ryu DY, Adegoke EO, Rahman MS, Park YJ, Pang MG (2022). Establishment of a male fertility prediction model with sperm RNA markers in pigs as a translational animal model. J Anim Sci Biotechnol.

[50] Holstein AFE, Knobil JD, Neill, editors. The Physiology of Reproduction. *Andrologia* 2009, 26(6):357–357.

[51] Xu W, Li H, Zhang N, Dong Z, Wang N, Shao C, Chen S (2016). Expression analysis and characterization of an autosome-localized tesk1 gene in half-smooth tongue sole (Cynoglossus semilaevis). Gene.

[52] Toshima J, Toshima JY, Amano T, Yang N, Narumiya S, Mizuno K (2001). Cofilin phosphorylation by protein kinase testicular protein kinase 1 and its role in integrin-mediated actin reorganization and focal adhesion formation. Mol Biol Cell.

[53] Bansal SK, Gupta N, Sankhwar SN, Rajender S (2015). Differential genes expression between fertile and infertile Spermatozoa revealed by Transcriptome Analysis. PLoS ONE.

[54] Ostermeier GC, Goodrich RJ, Moldenhauer JS, Diamond MP, Krawetz SA (2005). A suite of novel human spermatozoal RNAs. J Androl.

[55] Miller D, Ostermeier GC, Krawetz SA (2005). The controversy, potential and roles of spermatozoal RNA. Trends Mol Med.

[56] Johnson GA, Burghardt RC, Bazer FW, Seo H, Cain JW (2023). Integrins and their potential roles in mammalian pregnancy. J Anim Sci Biotechnol.

[57] Monkley SJ, Kostourou V, Spence L, Petrich B, Coleman S, Ginsberg MH, Pritchard CA, Critchley DR (2011). Endothelial cell talin1 is essential for embryonic angiogenesis. Dev Biol.

[58] Bridger PS, Haupt S, Leiser R, Johnson GA, Burghardt RC, Tinneberg HR, Pfarrer C (2008). Integrin activation in bovine placentomes and in caruncular epithelial cells isolated from pregnant cows. Biol Reprod.

[59] Zhao F, Liu H, Wang N, Yu L, Wang A, Yi Y, Jin Y (2020). Exploring the role of Luman/CREB3 in regulating decidualization of mice endometrial stromal cells by comparative transcriptomics. BMC Genomics.

[60] Lan X, Jin Y, Yang Y, Lin P, Hu L, Cui C, Li Q, Li X, Wang A (2013). Expression and localization of Luman RNA and protein during mouse implantation and decidualization. Theriogenology.

[61] Yoshie M, Tamura K, Hara T, Kogo H (2006). Expression of stathmin family genes in the murine uterus during early pregnancy. Mol Reprod Dev.

[62] Mota RR, Guimaraes SEF, Fortes MRS, Hayes B, Silva FF, Verardo LL, Kelly MJ, de Campos CF, Guimaraes JD, Wenceslau RR (2017). Genome-wide association study and annotating candidate gene networks affecting age at first calving in Nellore cattle. J Anim Breed Genet.

[63] Fortes MRS, Lehnert SA, Bolormaa S, Reich C, Fordyce G, Corbet NJ, Whan V, Hawken RJ, Reverter A (2012). Finding genes for economically important traits: Brahman cattle puberty. Anim Prod Sci.

[64] Magalhaes AF, de Camargo GM, Fernandes GAJ, Gordo DG, Tonussi RL, Costa RB, Espigolan R, Silva RM, Bresolin T, de Andrade WB (2016). Genome-Wide Association Study of Meat Quality Traits in Nellore cattle. PLoS ONE.

[65] Brunes LC, Baldi F, Lopes FB, Lobo RB, Espigolan R, Costa MFO, Stafuzza NB, Magnabosco CU (2021). Weighted single-step genome-wide association study and pathway analyses for feed efficiency traits in Nellore cattle. J Anim Breed Genet.

[66] Seabury CM, Oldeschulte DL, Saatchi M, Beever JE, Decker JE, Halley YA, Bhattarai EK, Molaei M, Freetly HC, Hansen SL (2017). Genome-wide association study for feed efficiency and growth traits in U.S. beef cattle. BMC Genomics.

[67] de Camargo GM, Costa RB, de Albuquerque LG, Regitano LC, Baldi F, Tonhati H (2015). Polymorphisms in TOX and NCOA2 genes and their associations with reproductive traits in cattle. Reprod Fertil Dev.

[68] Lee SH, Choi BH, Lim D, Gondro C, Cho YM, Dang CG, Sharma A, Jang GW, Lee KT, Yoon D (2013). Genome-wide association study identifies major loci for carcass weight on BTA14 in Hanwoo (Korean cattle). PLoS ONE.

[69] Srikanth K, Lee SH, Chung KY, Park JE, Jang GW, Park MR, Kim NY, Kim TH, Chai HH, Park WC (2020). A Gene-Set Enrichment and protein-protein Interaction Network-based GWAS with Regulatory SNPs identifies candidate genes and pathways Associated with carcass traits in Hanwoo Cattle. Genes (Basel).

[70] Bhuiyan MSA, Lim D, Park M, Lee S, Kim Y, Gondro C, Park B, Lee S (2018). Functional Partitioning of Genomic Variance and Genome-Wide Association Study for Carcass Traits in Korean hanwoo cattle using Imputed sequence level SNP data. Front Genet.

[71] Craig EA, Stevens MV, Vaillancourt RR, Camenisch TD (2008). MAP3Ks as central regulators of cell fate during development. Dev Dyn.

[72] Granados A, Alaniz VI, Mohnach L, Barseghyan H, Vilain E, Ostrer H, Quint EH, Chen M, Keegan CE (2017). MAP3K1-related gonadal dysgenesis: six new cases and review of the literature. Am J Med Genet Part C Seminars Med Genet.

[73] Das DK, Rahate SG, Mehta BP, Gawde HM, Tamhankar PM (2013). Mutation analysis of mitogen activated protein kinase 1 gene in Indian cases of 46,XY disorder of sex development. Indian J Hum Genet.

[74] Kimura E, Mongan M, Xiao B, Wang J, Carreira VS, Bolon B, Zhang X, Burns KA, Biesiada J, Medvedovic M et al. The role of MAP3K1 in the development of the Female Reproductive Tract. *bioRxiv* 2023:2023.2004.2020.537715.

[75] Chawala AR, Sanchez-Molano E, Dewhurst RJ, Peters A, Chagunda MGG, Banos G (2021). Breeding strategies for improving smallholder dairy cattle productivity in Sub-saharan Africa. J Anim Breed Genet.

[76] Moorey SE, Biase FH (2020). Beef heifer fertility: importance of management practices and technological advancements. J Anim Sci Biotechnol.

[77] Norling A, Hirschberg AL, Rodriguez-Wallberg KA, Iwarsson E, Wedell A, Barbaro M (2014). Identification of a duplication within the gene and novel candidate genes for primary ovarian insufficiency (POI) by a customized high-resolution array comparative genomic hybridization platform. Hum Reprod.

[78] Lo Turco EG, Souza GH, Garcia JS, Ferreira CR, Eberlin MN, Bertolla RP (2010). Effect of endometriosis on the protein expression pattern of follicular fluid from patients submitted to controlled ovarian hyperstimulation for in vitro fertilization. Hum Reprod.

[79] Wang S, Liao Y, Zhang H, Jiang Y, Peng Z, Ren R, Li X, Wang H (2022). Tcf12 is required to sustain myogenic genes synergism with MyoD by remodelling the chromatin landscape. Commun Biol.

[80] Fresch R, Courtney J, Brockway H, Wilson RL, Jones H (2023). HAND1 knockdown disrupts trophoblast global gene expression. Physiological Rep.

[81] Li X, Abdel-Moneim AE, Hu Z, Mesalam NM, Yang B (2022). Effects of chronic hypoxia on the gene expression profile in the embryonic heart in three Chinese indigenous chicken breeds (Gallus gallus). Front Veterinary Sci.

[82] Wu W, Kong X, Jia Y, Jia Y, Ou W, Dai C, Li G, Gao R (2022). An overview of PAX1: expression, function and regulation in development and diseases. Front Cell Dev Biol.

[83] Yamazaki Y, Urrutia R, Franco LM, Giliani S, Zhang K, Alazami AM, Dobbs AK, Masneri S, Joshi A, Otaizo-Carrasquero F et al. PAX1 is essential for development and function of the human thymus. Sci Immunol 2020, 5(44). 10.1126/sciimmunol.aax1036 PMC7189207 32111619

[84] Su D, Ellis S, Napier A, Lee K, Manley NR (2001). Hoxa3 and pax1 regulate epithelial cell death and proliferation during thymus and parathyroid organogenesis. Dev Biol.

[85] Sivakamasundari V, Kraus P, Sun W, Hu X, Lim SL, Prabhakar S, Lufkin T (2017). A developmental transcriptomic analysis of Pax1 and Pax9 in embryonic intervertebral disc development. Biology open.

[86] Takimoto A, Mohri H, Kokubu C, Hiraki Y, Shukunami C (2013). Pax1 acts as a negative regulator of chondrocyte maturation. Exp Cell Res.

[87] Cardoso Consentini CE, Wiltbank MC, Sartori R. Factors that Optimize Reproductive efficiency in dairy herds with an emphasis on timed Artificial Insemination Programs. Anim (Basel) 2021, 11(2). 10.3390/ani11020301 PMC7912388 33503935

[88] Medrano GA, Singh M, Plautz EJ, Good LB, Chapman KM, Chaudhary J, Jaichander P, Powell HM, Pudasaini A, Shelton JM et al. Mutant screen for reproduction unveils depression-associated Piccolo’s control over reproductive behavior. bioRxiv 2020:405985.

[89] Jeong J, Choi I. Effects of Alternative Splicing-Specific Knockdown of Tjp1 α + by Rbm47 on Tight Junctions Assembly during Blastocyst Development. *bioRxiv* 2023.

[90] Shivalingappa PKM, Sharma V, Shiras A, Bapat SA (2021). RNA binding motif 47 (RBM47): emerging roles in vertebrate development, RNA editing and cancer. Mol Cell Biochem.

[91] Mihalik J, Krehelova A, Kovarikova V, Solar P, Domorakova I, Pavliuk-Karachevtseva A, Hladova A, Rybarova S, Hodorova I. GPx8 expression in rat oocytes, embryos, and female genital organs during preimplantation period of pregnancy. Int J Mol Sci 2020, 21(17). 10.3390/ijms21176313 PMC7503774 32878231

[92] Purcell S, Neale B, Todd-Brown K, Thomas L, Ferreira MA, Bender D, Maller J, Sklar P, de Bakker PI, Daly MJ (2007). PLINK: a tool set for whole-genome association and population-based linkage analyses. Am J Hum Genet.

[93] Browning BL, Tian X, Zhou Y, Browning SR (2021). Fast two-stage phasing of large-scale sequence data. Am J Hum Genet.

[94] Bradbury PJ, Zhang Z, Kroon DE, Casstevens TM, Ramdoss Y, Buckler ES (2007). TASSEL: software for association mapping of complex traits in diverse samples. Bioinformatics.

[95] Wickham H. ggplot2: elegant graphics for data analysis. Springer International Publishing; 2016.

[96] Zhou X, Stephens M (2012). Genome-wide efficient mixed-model analysis for association studies. Nat Genet.

[97] R Core Team. R: A language and environment for statistical computing. In. Vienna, Austria: R Foundation for Statistical Computing; 2023.

[98] Dong SS, He WM, Ji JJ, Zhang C, Guo Y, Yang TL. LDBlockShow: a fast and convenient tool for visualizing linkage disequilibrium and haplotype blocks based on variant call format files. Brief Bioinform 2021, 22(4). 10.1093/bib/bbaa227 33126247

[99] Huang DW, Sherman BT, Tan Q, Collins JR, Alvord WG, Roayaei J, Stephens R, Baseler MW, Lane HC, Lempicki RA (2007). The DAVID Gene Functional classification Tool: a novel biological module-centric algorithm to functionally analyze large gene lists. Genome Biol.

[100] Xie C, Mao X, Huang J, Ding Y, Wu J, Dong S, Kong L, Gao G, Li CY, Wei L (2011). KOBAS 2.0: a web server for annotation and identification of enriched pathways and diseases. Nucleic Acids Res.

