# Supplementary material for: Genome-wide association study identifies genomic regions associated with key reproductive traits in Korean Hanwoo cows
Source: BMC Genomics. 2024 May 23;25:496. doi: 10.1186/s12864-024-10401-3 (PMC11112828; doi:10.1186/s12864-024-10401-3)
Supplement: Supplementary file 1 — Additional file 1: Fig S1. Regional association plot showing the distribution of significant loci associated with AFC, CI, GL, and NAIPC at various BTA (top), and heatmap of LD (bottom). Description: The red horizontal line indicates -log10P = 4.30 [file 12864_2024_10401_MOESM1_ESM.pdf]

Regional association plot and LD heatmap of BTA8 region for AFC

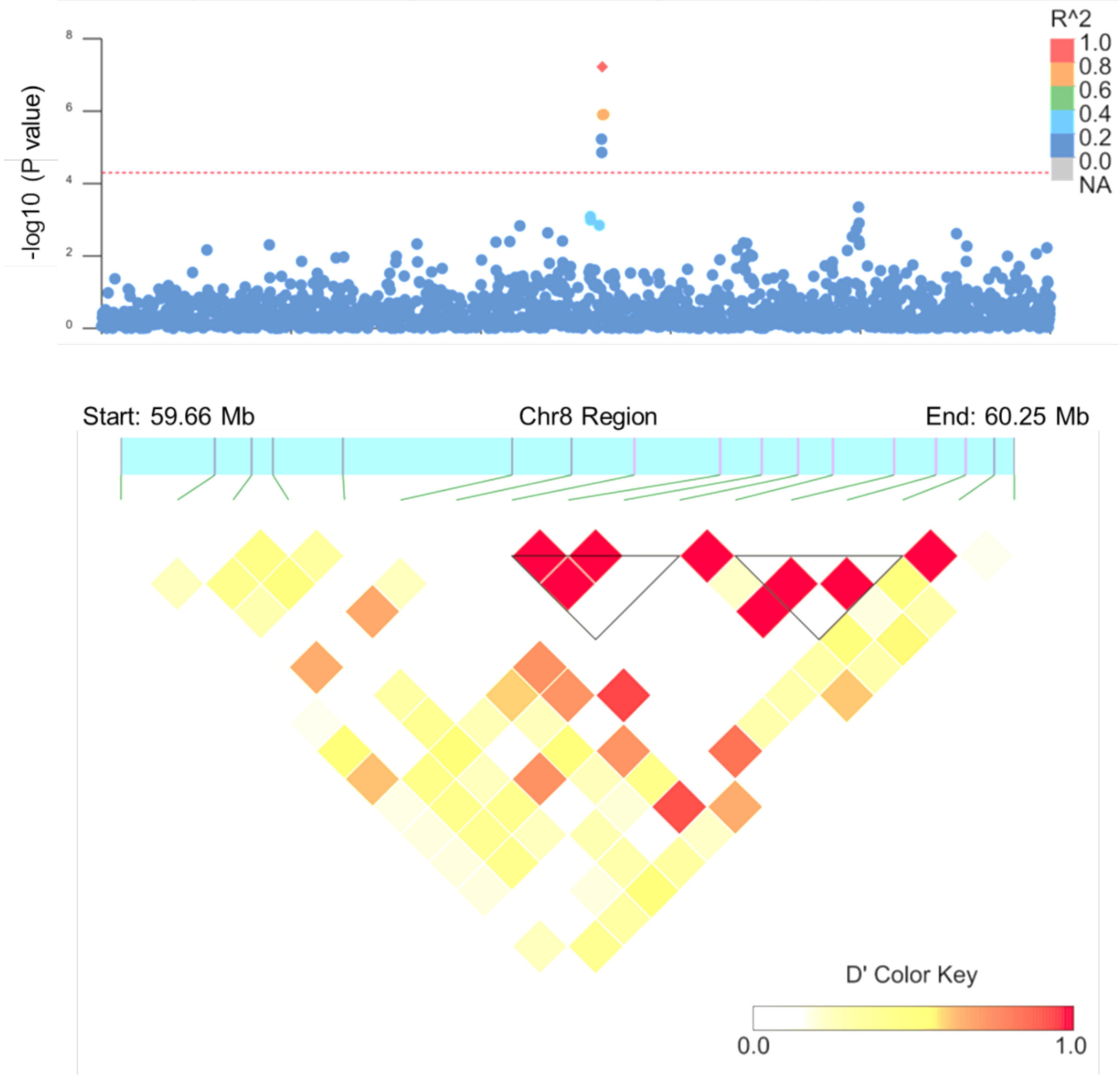

Regional association plot and LD heatmap of BTA7 region for CI

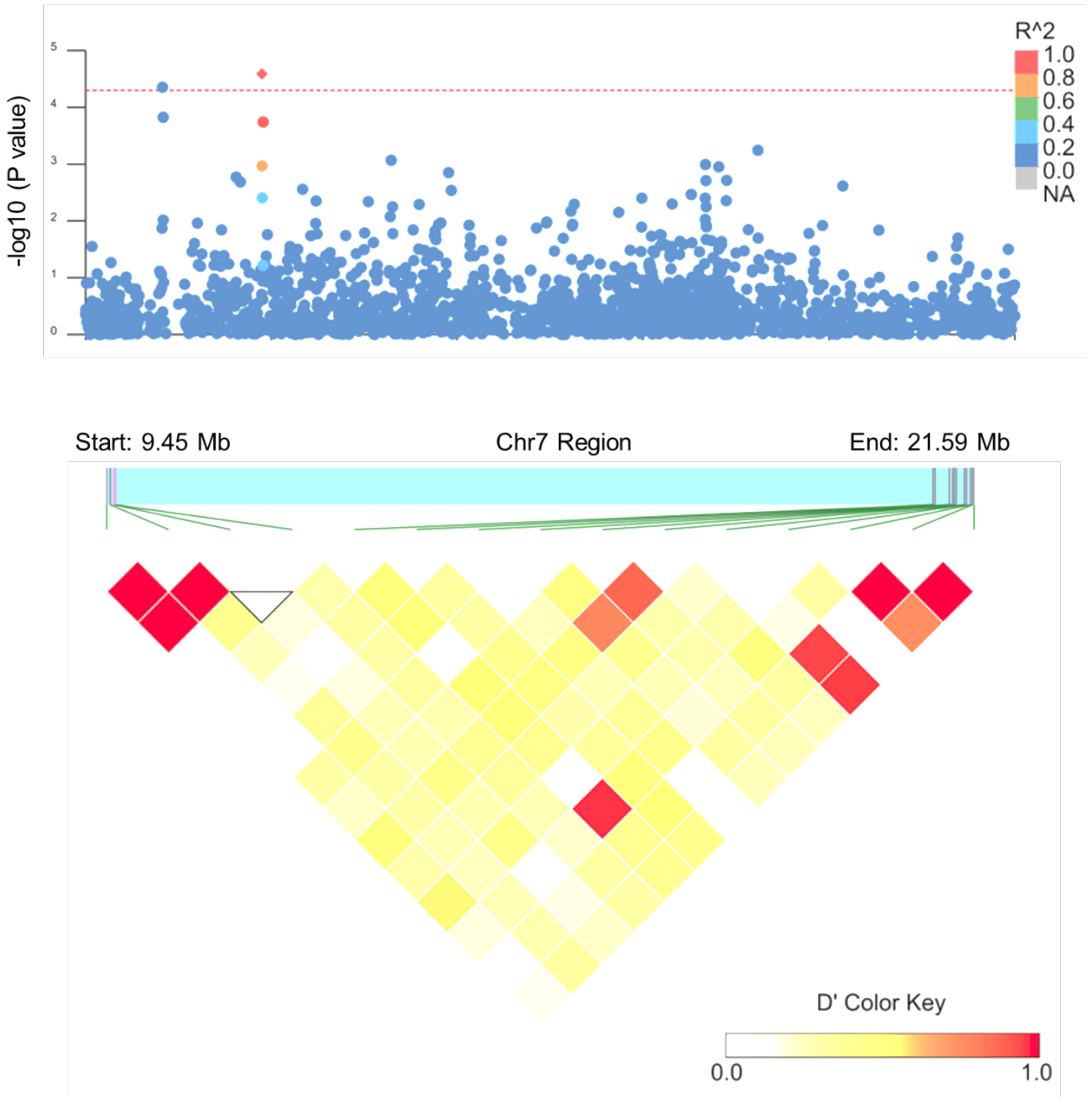

Regional association plot and LD heatmap of BTA10 region for CI

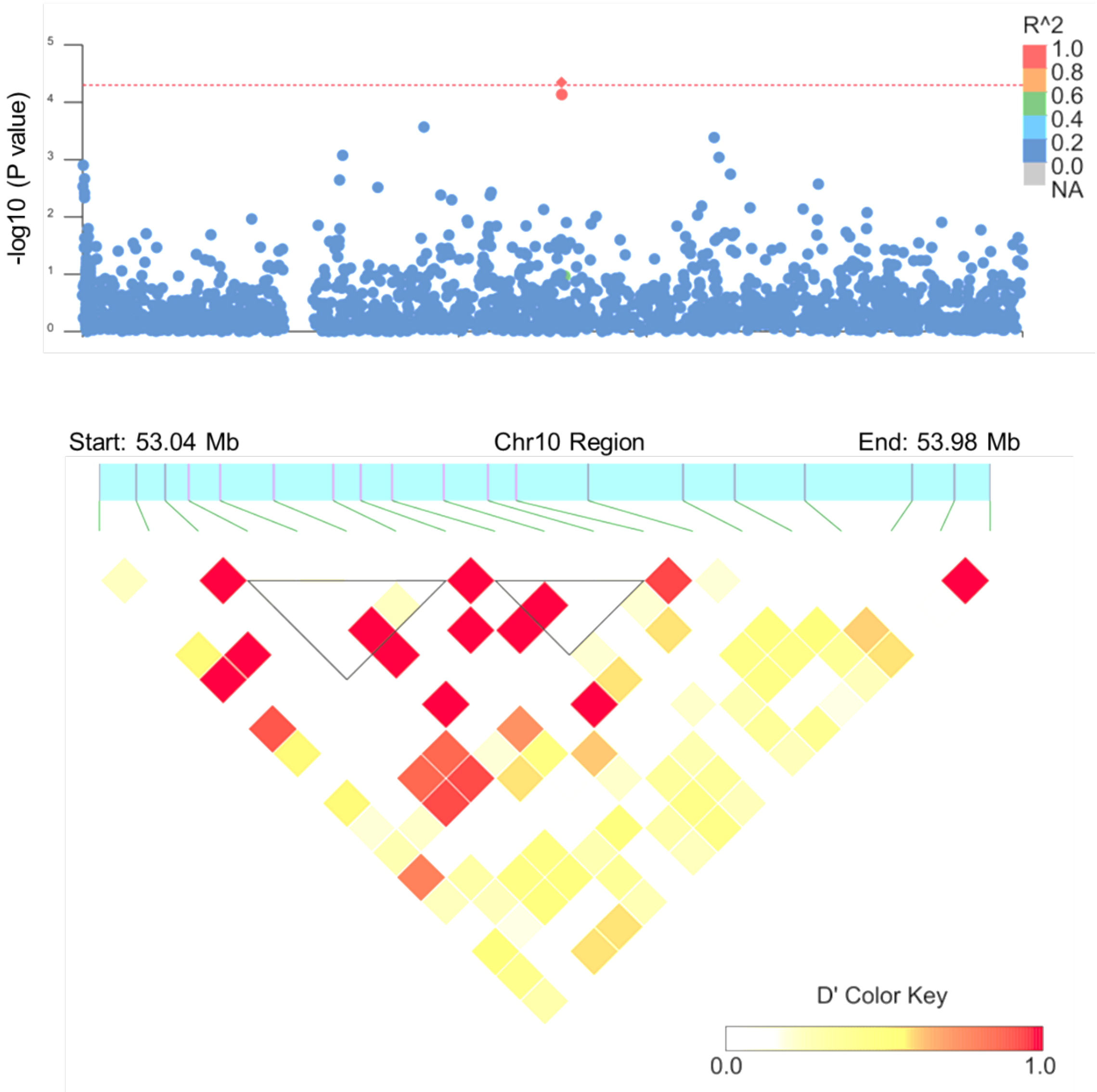

Regional association plot and LD heatmap of BTA17 region for CI

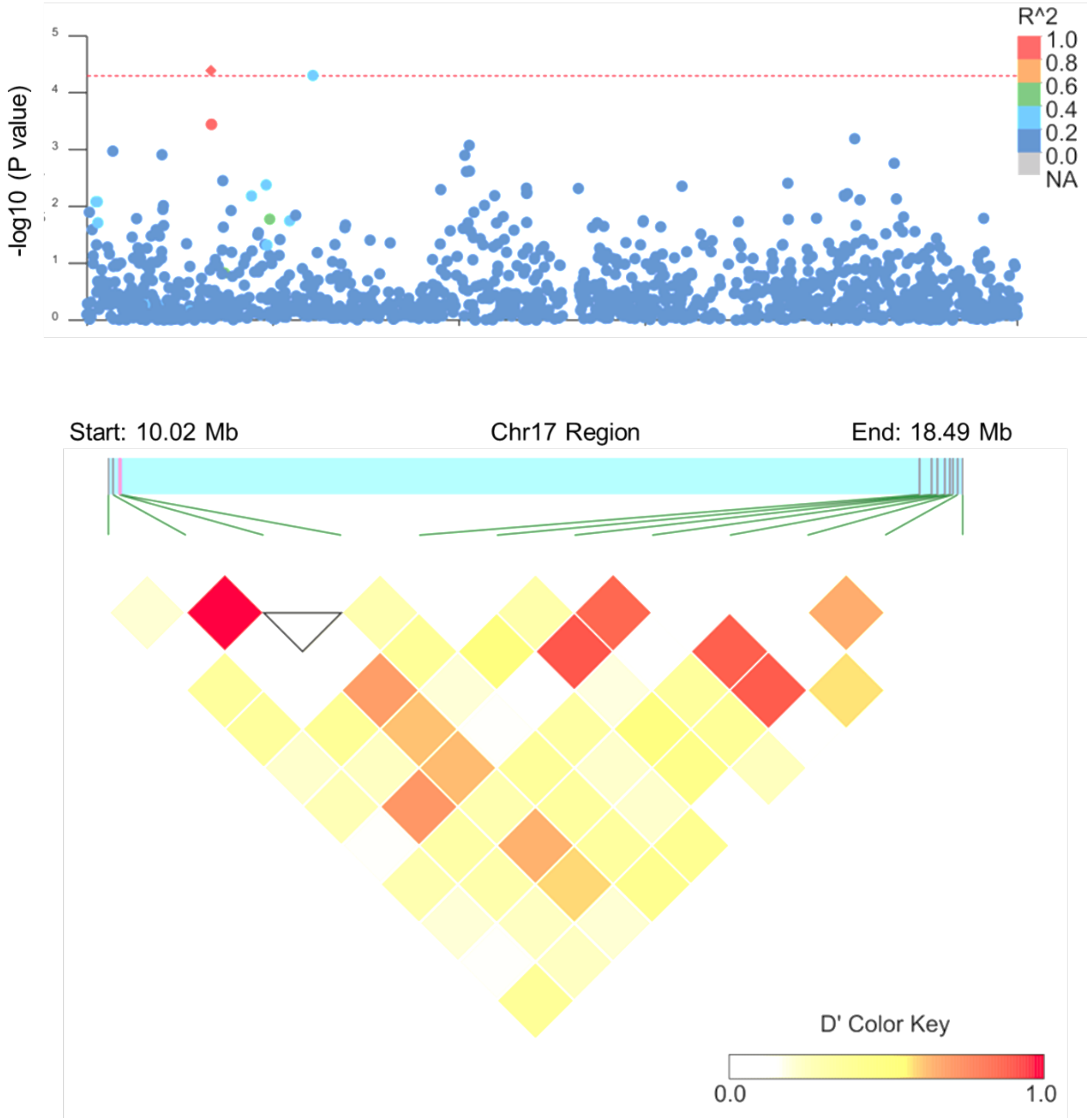

Regional association plot and LD heatmap of BTA7 region for GL

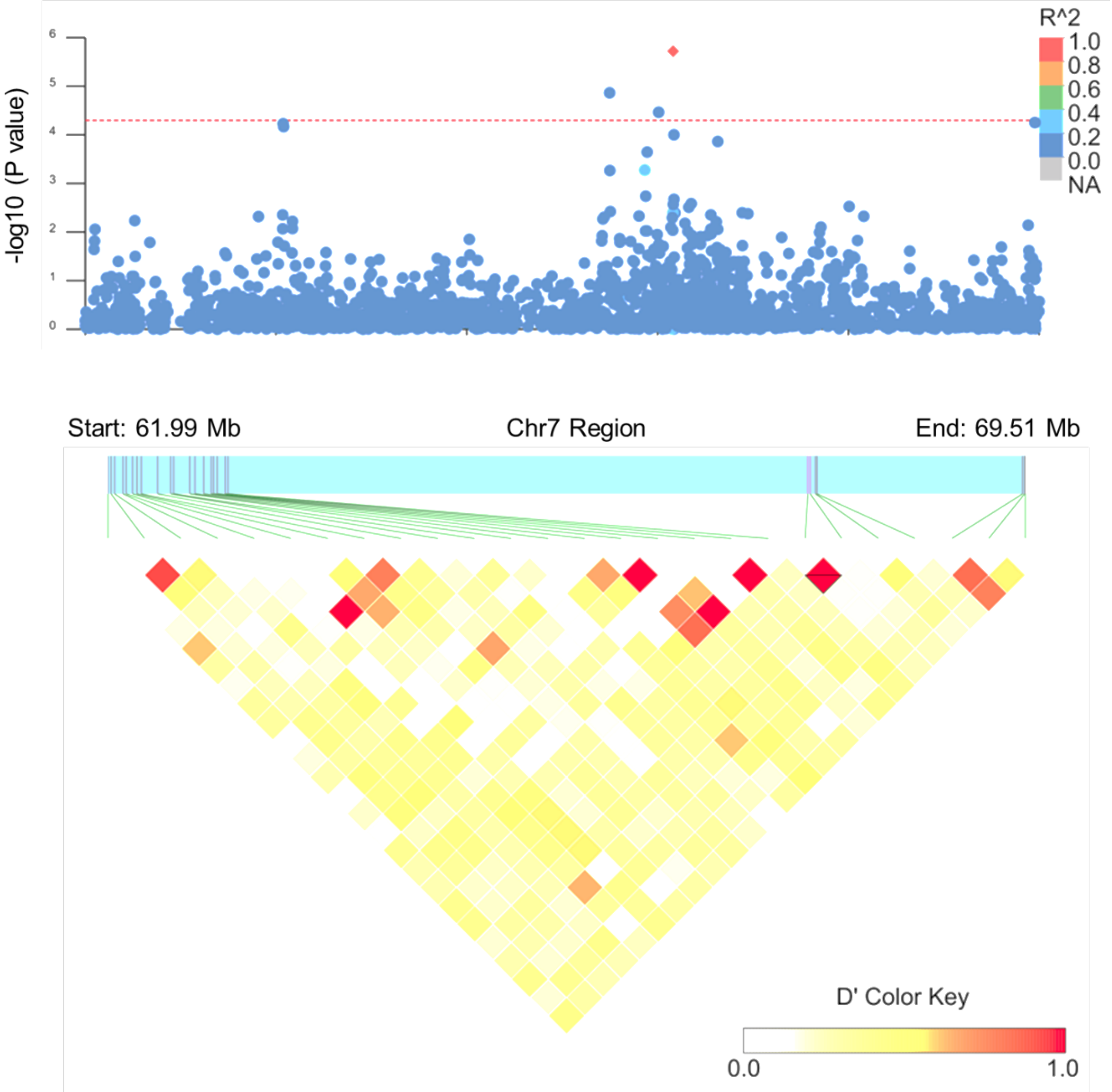

Regional association plot and LD heatmap of BTA10 region for GL

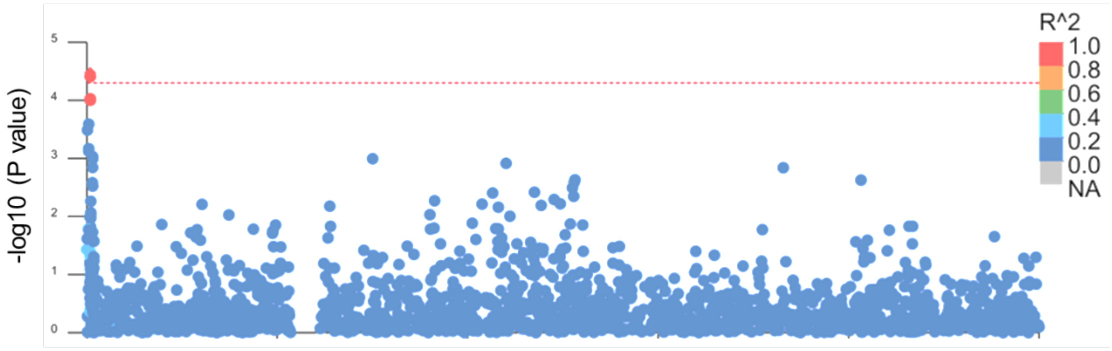

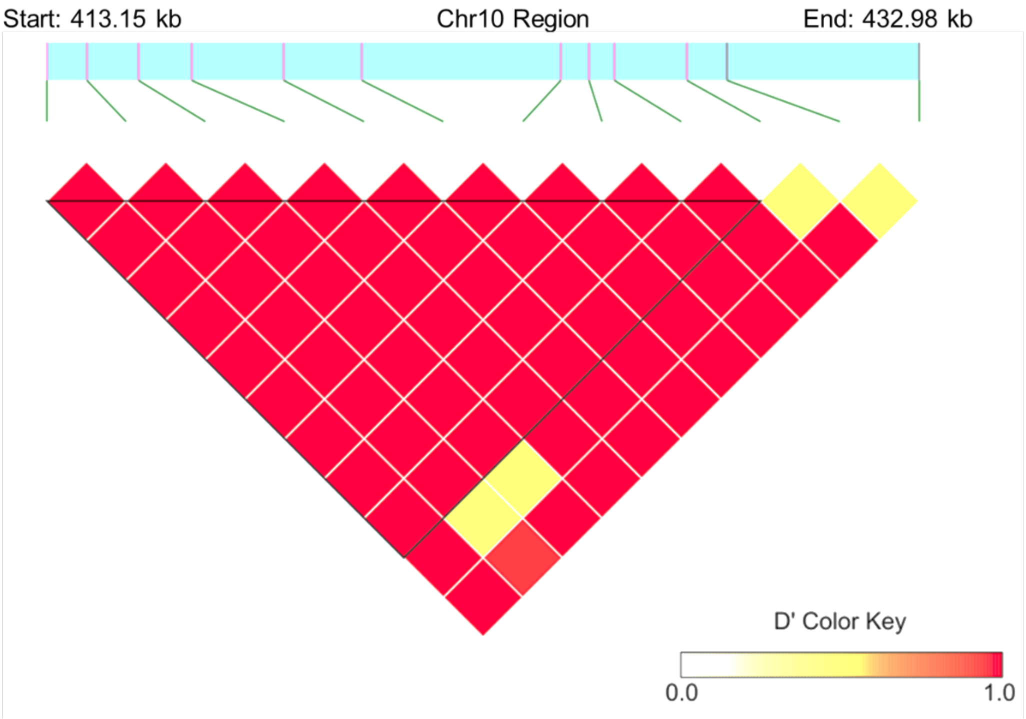

Regional association plot and LD heatmap of BTA13 region for GL

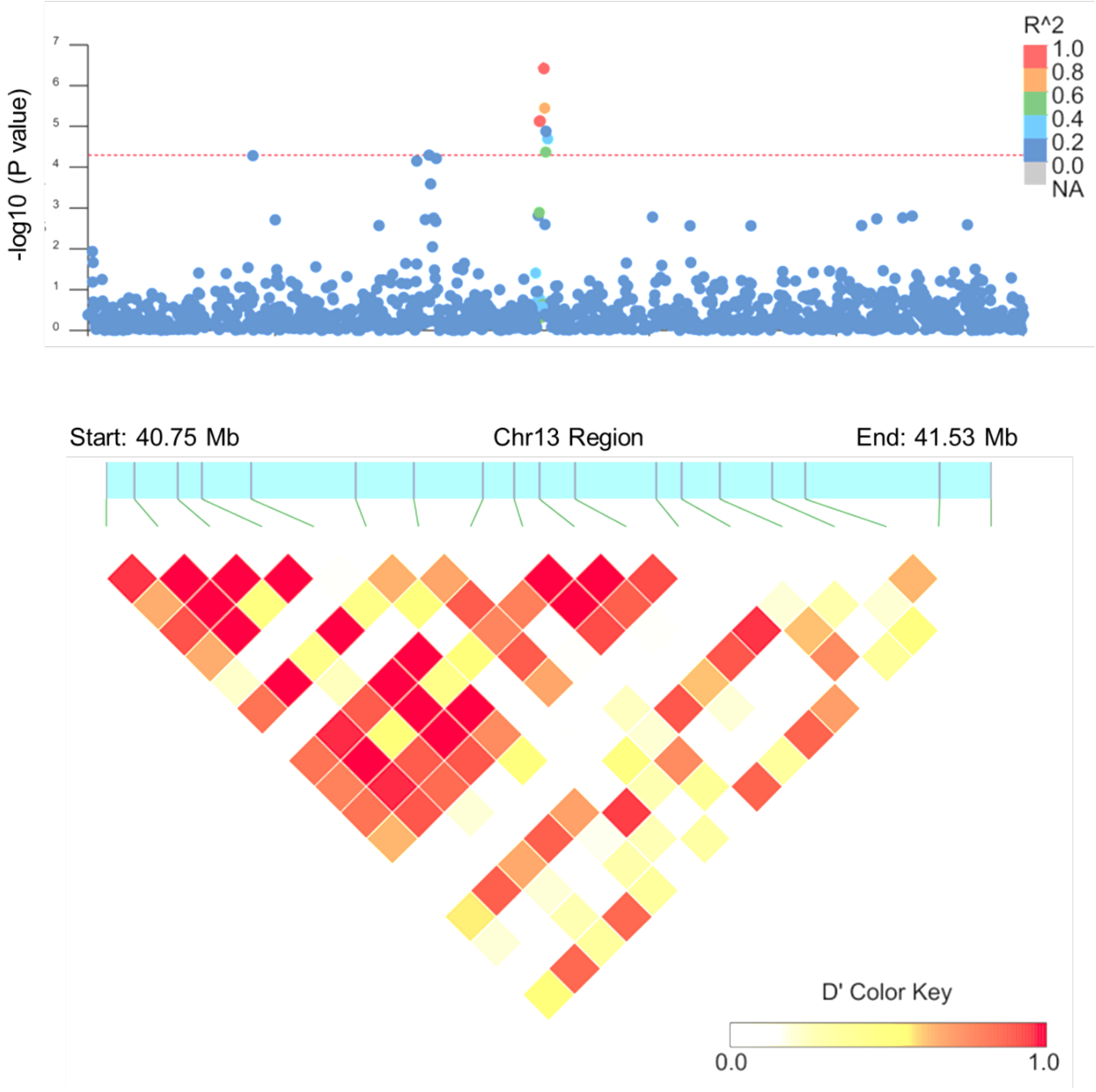

Regional association plot and LD heatmap of BTA6 region for NAIPC

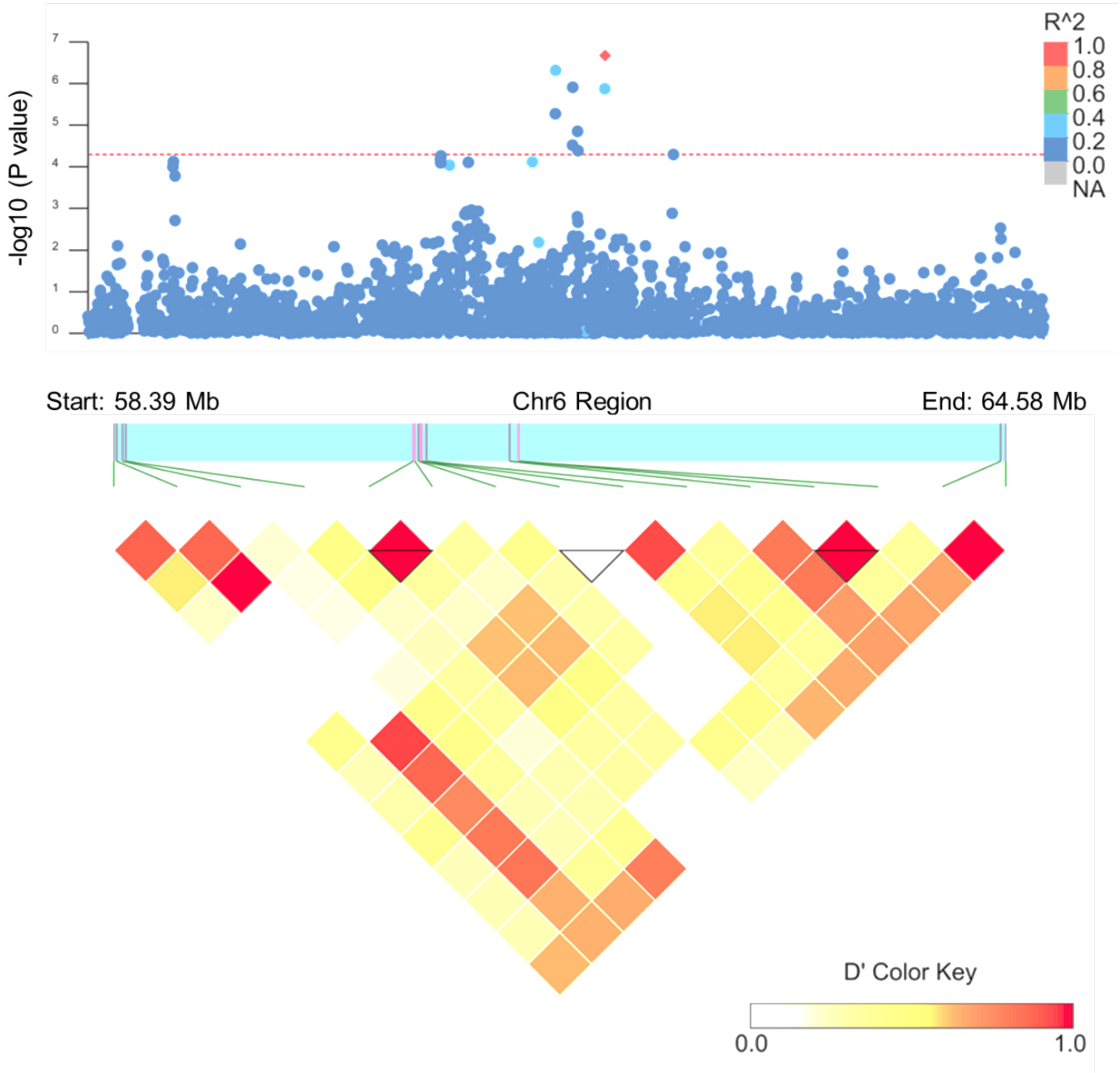

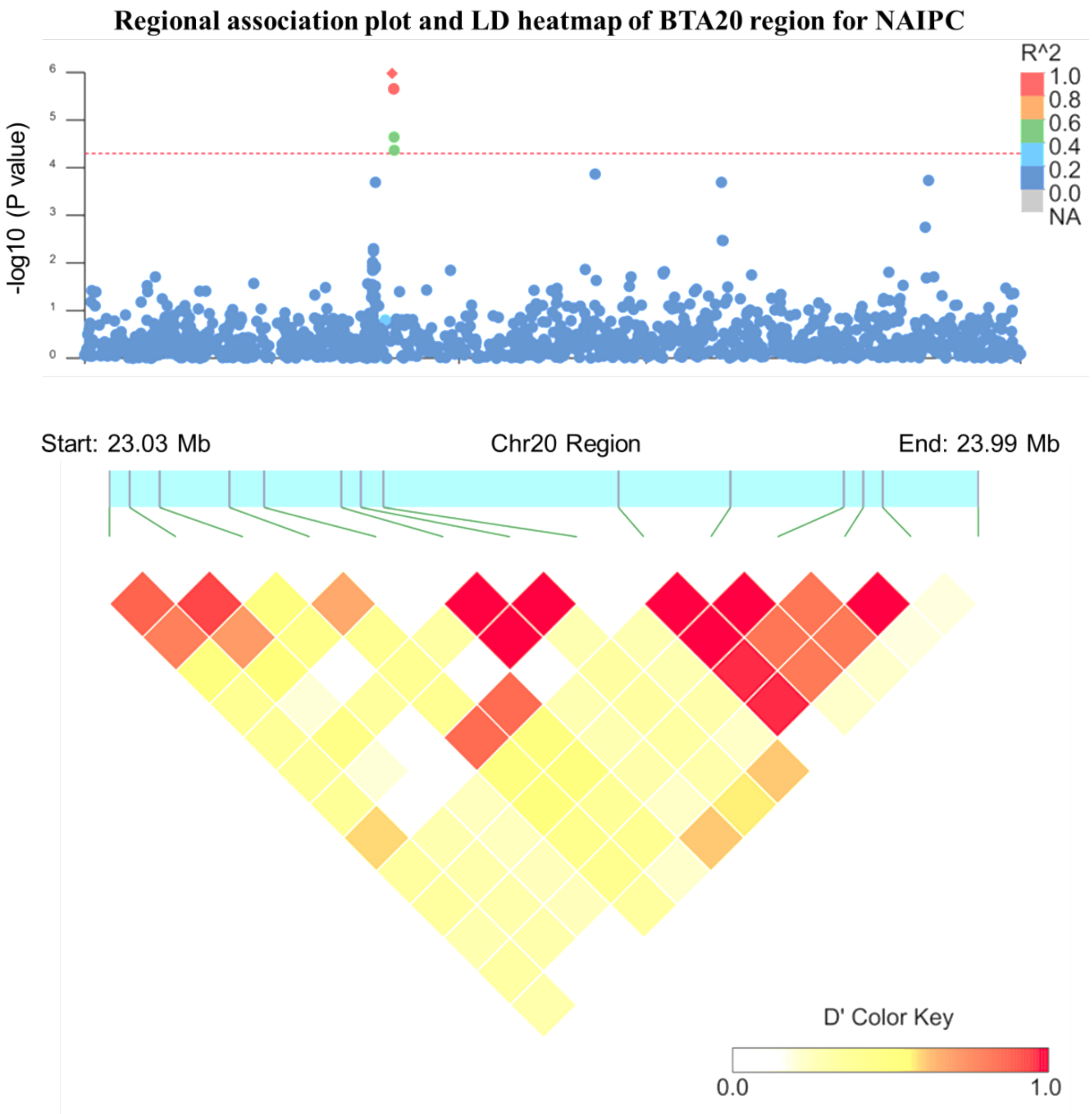

Fig. S1 Regional association plot showing the distribution of significant loci associated with AFC, CI, GL, and NAIPC at various BTA (top) and heatmap of LD (bottom). The red horizontal line indicates -log10P = 4.30.
