# Supplementary material for: Genome-wide association study identifies genomic regions associated with key reproductive traits in Korean Hanwoo cows
Source: BMC Genomics. 2024 May 23;25:496. doi: 10.1186/s12864-024-10401-3 (PMC11112828; doi:10.1186/s12864-024-10401-3)
Supplement: Supplementary file 2 — Additional file 2: Fig S2 The quantile-quantile (QQ) plots and genomic inflation factor (λ) of the GWAS analysis for reproductive traits in Korean Hanwoo cows. Description: QQ plots showing late separation between the observed and expected p-values (-log10P). The genomic inflation factor (λ) is close to 1, indicating that there is no population stratification [file 12864_2024_10401_MOESM2_ESM.pdf]

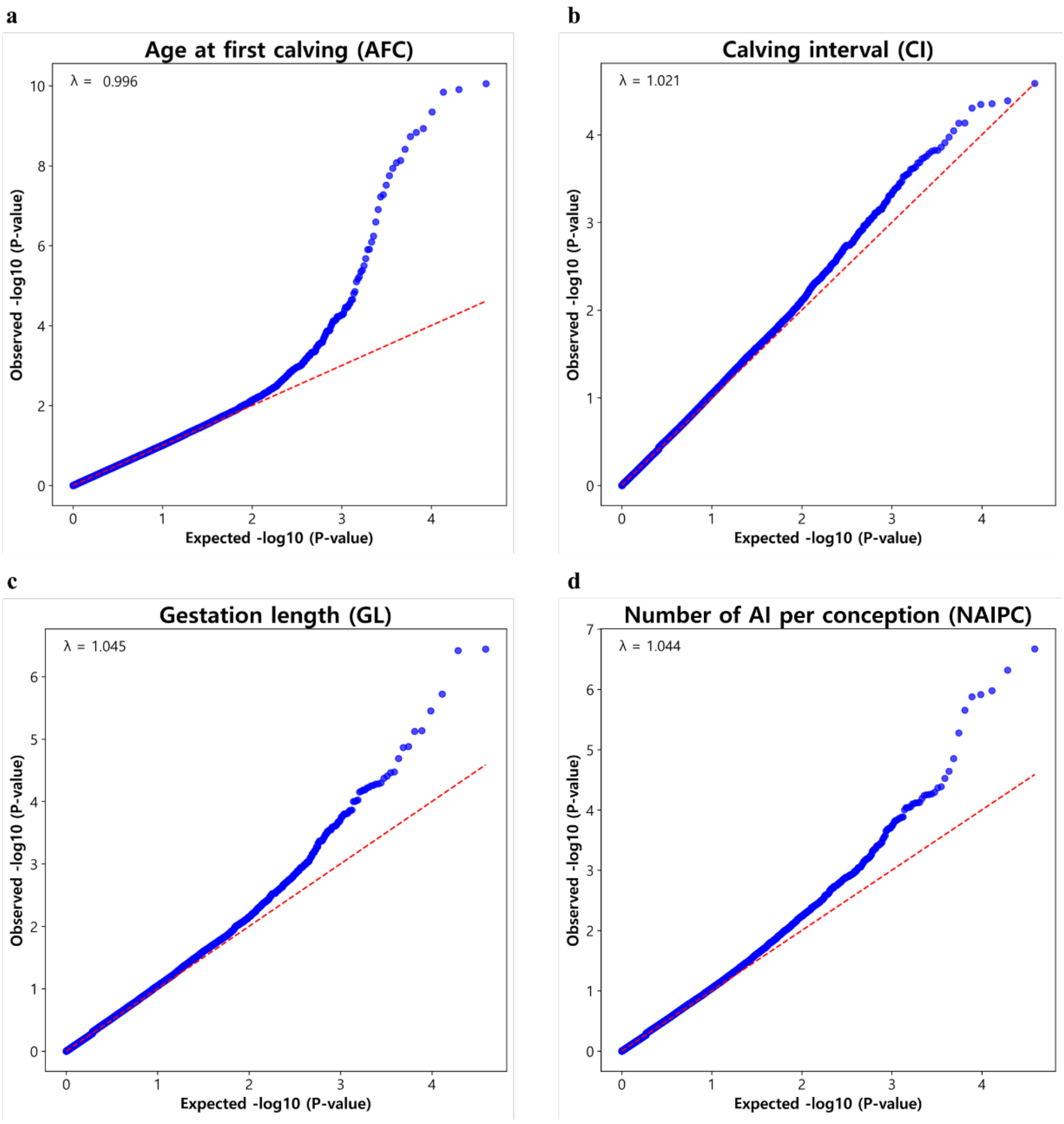

Fig. S2 The quantile-quantile (QQ) plots and genomic inflation factor (λ) of the GWAS analysis for reproductive traits in Korean Hanwoo cows. Description: QQ plots showing late separation between the observed and expected p-values (-log10P). The genomic inflation factor (λ) is close to 1, indicating that there is no population stratification.
