# Supplementary material for: Genome-wide association study identifies genomic regions associated with key reproductive traits in Korean Hanwoo cows
Source: BMC Genomics. 2024 May 23;25:496. doi: 10.1186/s12864-024-10401-3 (PMC11112828; doi:10.1186/s12864-024-10401-3)
Supplement: Supplementary file 3 — Additional file 3: Fig S3. Bar plot of -log10 of the p-values of selected GO terms. Description: This file provides the GO term (p < 0.05) plot of molecular functions, cellular components, and KEGG pathways [file 12864_2024_10401_MOESM3_ESM.pdf]

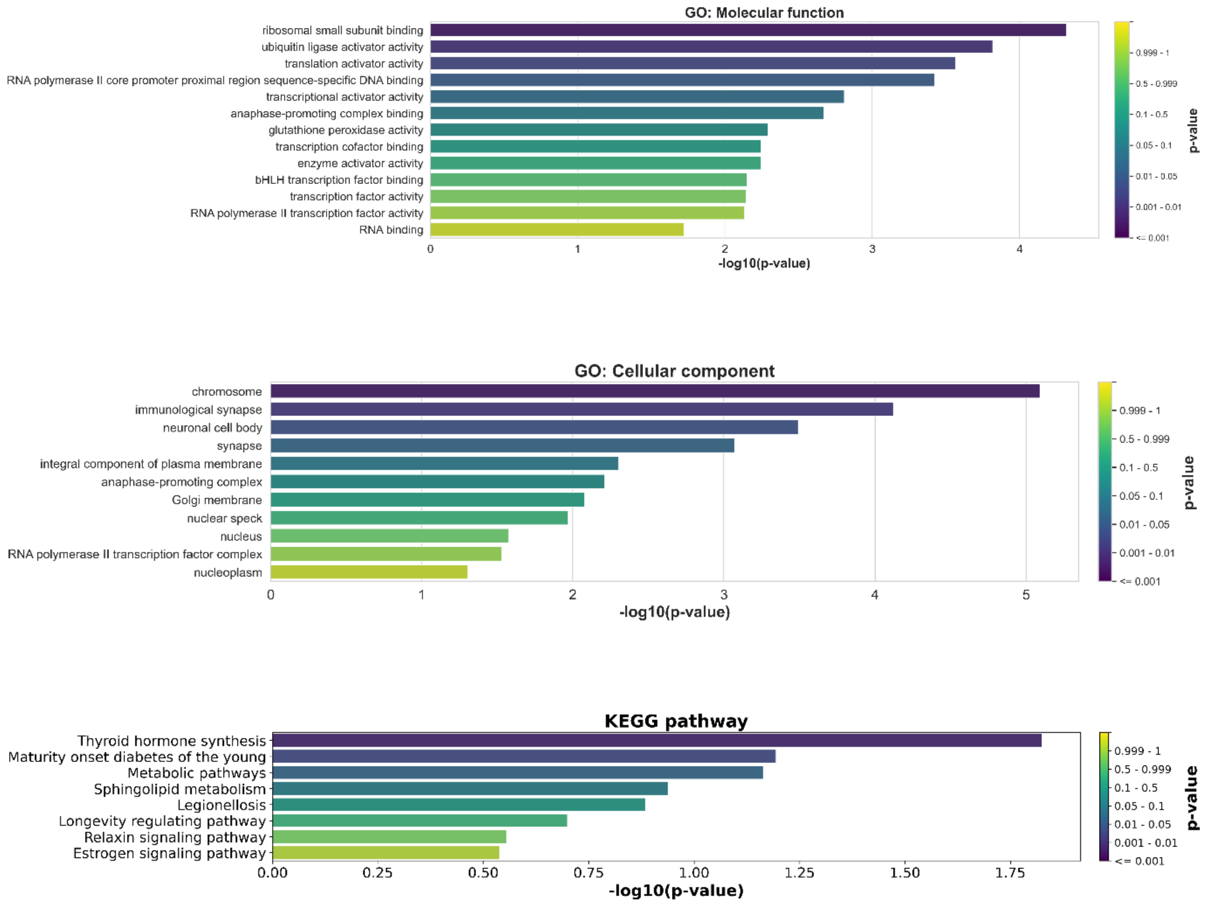

Fig. S3 Bar plot of -log10 of the p-values of selected GO terms. Description: This file provides the GO term ($p < 0.05$) plot of molecular functions, cellular components, and KEGG pathways.
